# Systemic inflammation response index as a clinical outcome evaluating tool and prognostic indicator for hospitalized stroke patients: a systematic review and meta-analysis

**DOI:** 10.1186/s40001-023-01446-3

**Published:** 2023-11-01

**Authors:** Yong-Wei Huang, Ye Zhang, Cui Feng, Yin-Hua An, Zong-Ping Li, Xiao-Shuang Yin

**Affiliations:** 1 Department of Neurosurgery, Mianyang Central Hospital, School of Medicine, University of Electronic Science and Technology of China, Mianyang, Sichuan China; 2 Department of Ultrasound, Mianyang Central Hospital, School of Medicine, University of Electronic Science and Technology of China, Mianyang, Sichuan China; 3 Center of Reproductive Medicine, Mianyang Central Hospital, School of Medicine, University of Electronic Science and Technology of China, Mianyang, Sichuan China; 4 Department of Immunology, Mianyang Central Hospital, School of Medicine, University of Electronic Science and Technology of China, Mianyang, Sichuan China

**Keywords:** Systemic inflammation response index, Stroke, Intracerebral hemorrhage, Subarachnoid hemorrhage, Clinical outcome

## Abstract

**Background:**

Stroke, which is the main element of cerebrovascular disease (CVD), has become the foremost reason for death and disability on a global scale. The systemic inflammation response index (SIRI), a newly developed and comprehensive indicator, has demonstrated promise in forecasting clinical results for diverse ailments. Nevertheless, the uncertainty surrounding the assessment and prediction of clinical outcomes for stroke patients by SIRI persists, and the conflicting findings from the limited studies conducted on this matter further complicate the situation. Consequently, we performed a thorough systematic review and meta-analysis to explore the correlation between SIRI and the clinical results in individuals suffering from stroke.

**Methods:**

This research was registered in PROSPERO and carried out following the PRISMA guidelines. A thorough investigation was carried out on PubMed, Embase, the Cochrane Library, Web of Science, and Scopus databases. Furthermore, we conducted a manual search in Chinese databases, such as China national Knowledge Infrastructure (CNKI), WanFang, VIP, and China Biology Medicine (CBM). We assessed the potential for bias in the studies included by utilizing the Newcastle–Ottawa Scale (NOS) tool. Adverse clinical outcomes were the main focus of the study, with secondary endpoints including mortality, the predictive value of SIRI, SIRI values across various endpoints, and clinical parameters associated with subarachnoid hemorrhage (SAH) in relation to low and high SIRI group.

**Results:**

Following rigorous evaluation, a grand total of 22 investigations, encompassing a populace of 12,737 individuals, were considered suitable for incorporation in the final analysis. The findings from our meta-analysis indicate a strong and consistent correlation between elevated SIRI levels and adverse functional outcomes, irrespective of the method used to evaluate unfavorable outcomes. Furthermore, increased SIRI values have a strong correlation with mortality rates in both the short and long term. Besides, SIRI is a useful indicator of the severity of SAH. SIRI demonstrates strong predictive ability in identifying unfavorable outcomes and stroke-related pneumonia (SAP), as higher SIRI values are typically linked to negative endpoints. Nevertheless, the meta-analysis indicated that there was no significant increase in the risk of early neurological deterioration (END) and acute hydrocephalus (AHC) in high SIRI group when comparing to low SIRI.

**Conclusion:**

This study could potentially pave the way for groundbreaking insights into the relationship between SIRI and stroke patient outcomes, as it appears to be the first meta-analysis to explore this association. Given the critical role of the inflammatory response in stroke recovery, closely monitoring patients with high SIRI levels could represent a promising strategy for mitigating brain damage post-stroke. Thus, further investigation into SIRI and its impact on clinical outcomes is essential. While our initial findings offer valuable insights into this area, continued research is necessary to fully elucidate the potential of SIRI, ideally through dynamic monitoring and large-scale, multi-center studies. Ultimately, this research has the potential to inform clinical decision-making and improve patient outcomes following stroke.

**Systematic review registration:** https://www.crd.york.ac.uk/prospero/; Identifier CRD42023405221.

**Supplementary Information:**

The online version contains supplementary material available at 10.1186/s40001-023-01446-3.

## Introduction

Studies in epidemiology have shown that the prevalence of cerebrovascular disease (CVD) has exceeded that of heart disease, emerging as the primary factor for death and impairment in the adult population [1, 2]. The occurrence of stroke is increasing as it is the main element of CVD. Accounting for 84.4% of all strokes, ischemic stroke (IS) is a prevalent sub-type [3]. Hemorrhagic stroke (HS), a more severe sub-type, consists of intracerebral hemorrhage (ICH) and subarachnoid hemorrhage (SAH). ICH experiences an annual increase of 3.41 million cases [4], while SAH contributes to 5% of total stroke cases [5]. Both IS and HS result in elevated mortality rates and prolonged disability [6–8]. With the population getting older, there will be a substantial rise in the burden of stroke in the coming years. Hence, it is imperative to create a straightforward, user-friendly, economical indicator that can anticipate the likelihood of unfavorable results and offer supplementary details grounded in clear pathophysiological principles for subsequent treatment. Since blood routine tests are essential for every admitted patient, a new indicator that relies on the absolute values of blood cell counts demonstrates potential.

The SIRI, an innovative and comprehensive indicator, relies on the absolute counts of neutrophils, monocytes, and lymphocytes (N × M/L) in the peripheral blood as a measure. The body’s inflammatory status can be more comprehensively reflected by these three blood cells, which represent distinct pathways of inflammation and immunity, as compared to peripheral blood cell ratios like neutrophil/lymphocyte ratio (NLR), lymphocyte/monocyte ratio (LMR), and platelet/lymphocyte ratio (PLR) [9–12]. Previous studies have extensively utilized SIRI to evaluate the regression of tumor patients and forecast unfavorable clinical treatment regression in pancreatic, gastric, and hepatocellular cancers [13]. Moreover, research has indicated that SIRI additionally mirrors the extent of atherosclerosis and forecasts the medical results in individuals with ICH, SAH, and those receiving intravascular mechanical thrombectomy for large artery occlusive stroke [14–16]. In patients with rheumatoid arthritis, there has been a connection between SIRI and the potential for developing acute ischemic stroke (AIS) [17]. Nevertheless, despite certain research indicating that SIRI holds promise as a valuable instrument for diagnosing and forecasting results in individuals with stroke, its ability to anticipate functional outcomes in stroke patients is restricted, and the results are contradictory, leaving the connection between SIRI and clinical outcomes uncertain. Hence, we conducted a comprehensive review and meta-analysis to investigate the correlation between SIRI and the clinical results in individuals affected by stroke.

## Methods

### Search strategy

The systematic review and meta-analysis followed the PRISMA guidelines [18] and was registered on PROSPERO with the identifier CRD42023405221 (https //www.crd.york.ac.uk/PROSPERO/) [19]. Additional file 1: Table S1 contains the PRISMA checklist. PubMed was searched using the keywords (“Systemic inflammation response index” OR “System inflammation response index” OR “Systemic inflammatory response index” OR “SIRI”) AND (“Patients”). We used the identical search approach for Embase, Cochrane Library, Web of Science, and Scopus. Furthermore, we conducted a manual search in Chinese databases, such as China national Knowledge Infrastructure (CNKI), WanFang, VIP, and China Biology Medicine (CBM). To minimize selection bias, articles in both English and Chinese were taken into account during the search, which spanned from the beginning to February 12, 2023. Additional file 1: Table S2 presents the detailed search strategy.

### Study selection

We included studies that satisfied the following PICO criteria: (1) Population: individuals who have experienced a stroke, including IS and HS (ICH and SAH); (2) Intervention: mechanical thrombectomy, intravenous thrombolysis, surgical procedures (coiling or clipping), conservative treatment, or no treatment; (3) Comparisons: low SIRI vs. high SIRI; evaluating different SIRI values at different endpoints; (4) Outcomes: functional outcomes (measured by modified Rankin Scale [mRS] or Glasgow Outcome Score [GOS] at follow-up), mortality, predictive value of SIRI, SIRI values between poor and good outcomes, stroke-associated pneumonia (SAP) and non-SAP, early neurological deterioration (END) and non-END; SAH-associated clinical parameters between high SIRI and low SIRI, including Hunt-Hess Scale (HHS), modified Fisher Scale (mFS), delayed cerebral ischemia (DCI), vasospasm, and acute hydrocephalus (AHC). We did not include reviews, editorials, commentaries, case reports, letters to the editor, systematic reviews and meta-analyses, notes, replies, and conference abstracts because these types of records are insufficient for data.

Both reviewers (H Y-W and Z Y) individually examined the titles and abstracts of all the records that were obtained. Two reviewers independently assessed the relevant studies in their entirety and made decisions on article inclusion or exclusion according to the eligibility criteria. In case of discordance, the corresponding authors (L Z-P and Y X-S) would adjudicate.

### Data extraction

Data were independently extracted into separate Excel spreadsheets by two reviewers, namely F C and A Y-H. To ensure accuracy, the source material and the spreadsheets were cross-checked with each other. Data collection included the first author's name, year of publication, country, study design, sample size, age, range, gender, stroke type, intervention type, SIRI cutoff (× 10^9^/L), primary and secondary endpoints, as well as the duration of follow-up. If any discrepancies were found, they were resolved by the corresponding author (L Z-P and Y X-S).

### Study outcomes

The primary outcome of this study was the assessment of functional outcomes, as measured by the mRS or GOS at follow-up. The definition of mRS and GOS is presented in Additional file 1: Table S3. The secondary outcomes included mortality, the predictive value of SIRI, SIRI values between poor/good outcomes, the SAP/non-SAP, and END/non-END. Additionally, the study analyzed the differences in HHS, mFS, DCI, vasospasm, and AHC between patients with low SIRI and high SIRI.

### Bias assessment

Two independent reviewers (H Y-W and F C) assessed the risk of bias of the included studies using the Newcastle–Ottawa Scale (NOS) tool [20] in a blind manner. The risk of bias summaries was then cross-checked, and any unresolved discrepancies were resolved by the corresponding author (LZ-P and YX-S).

### Statistical analysis

We computed odds ratios (ORs) and their corresponding 95% confidence intervals (CIs) for binary variables. Continuous variables were used to calculate the mean difference (MD) along with their corresponding 95% CIs. If there is a substantial difference in the values of continuous variables, we employed the standard mean difference (SMD) for conducting meta-analysis. We extracted ORs and their corresponding 95% CIs from studies that had adjusted for confounding factors. The mean and standard deviation (SD) were estimated by utilizing the sample size, median, and interquartile range. These estimates were obtained using the optional estimation techniques described in McGrath et al.’s publication [21], which can be accessed at https://smcgrath.shinyapps.io/estmeansd/. To consider the variation in clinical characteristics, we performed meta-analyses and subgroup analyses utilizing the random-effects approach if the heterogeneity exceeds 50%, or the fixed-effects approach if the heterogeneity is less than 50% [22]. When there were more than five studies included, subgroups analyses were conducted based on the sub-type stroke. Significant heterogeneity was assessed by conducting the Cochrane *Q* test (*P* < 0.1 or *I*^2^ > 50%) [23]. Statistical significance was determined using a significance level of *P* < 0.05. Funnel plots were utilized to evaluate publication bias. The statistical analyses were conducted using Review Manager software (version 5.3.3), which can be found at https://training.cochrane.org/online-learning/core-softwarecochrane-reviews/revman.

## Results

### Study selection

**Fig. 1:**
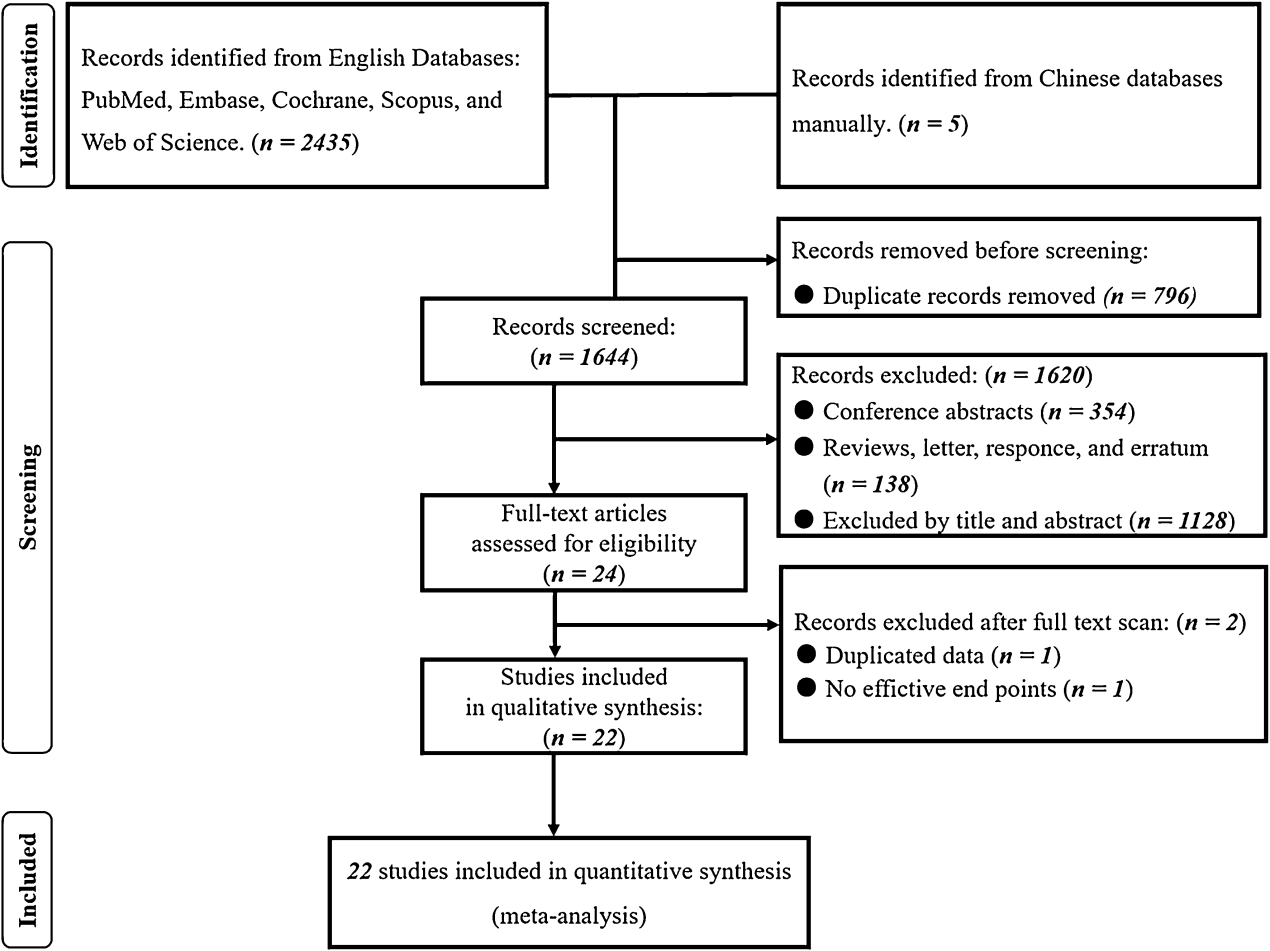
PRISMA flowchart of included studies

We acquired a total of 2435 publications using the search method on June 30, 2023. After eliminating 796 duplicates, we evaluated the remaining 1644 publications by their article type, title, and abstracts and we excluded 1620 publications that were not relevant. We thoroughly reviewed the remaining 24 publications for potential eligibility [9, 10, 14–16, 24–42]. Two studies [15, 40] shared almost the same data and were from the same author; thus, we combined the data and treated them as a single study. The exclusion of a study [35] was based on the absence of sufficient endpoints. In this systematic review and meta-analysis (Fig. 1), a total of 22 studies [9, 10, 14–16, 24–34, 36–39, 41, 42] were ultimately incorporated.

### Characteristics of the included studies

The 22 studies included in this systematic review and meta-analysis were published between 2020 and 2023. Among them, 5 articles were prospective studies [25, 29, 31, 33, 42], and the remaining 17 articles [9, 10, 14–16, 24, 26–28, 30, 32, 34, 36–39, 41] were retrospective studies. The studies were conducted in China (*n* = 19, two studies were from the MIMIC database), Italy (*n* = 1), and Korea (*n* = 2), with a total of 12,931 patients included. Two research studies employed a 1:1 propensity score matching (PSM) technique to equalize the impact of potential confounders, leading to the incorporation of 12,737 individuals in the analysis. 11 studies [10, 16, 24–26, 28–30, 32, 34, 39] focused on AIS, 11 studies focused on HS including 5 studies [9, 31, 33, 36, 42] focused on ICH, and 6 studies [14, 15, 27, 37, 38, 41] focused on SAH. The SIRI cutoff range was between 0.77 and 6.48 (× 10^9^/L), while the duration of follow-up varied from hospitalization to one year post-discharge. Table 1 provides a summary of the findings from the studies that were included.

**Table 1:** The baseline characteristics of included studies

Author	Year	Nation	Study design	Participants (*n*)	Male (%)	Age (y)	Type of stroke	Type of intervention	Cutoff (× 10^9^/L)	Primary endpoints	Secondary endpoints	Follow-up	NOS
Fei et al.	2020	China	Retrospective cohort study	146	63.01	60.21 ± 13.84	ICH in basal ganglia	–	5.4	END (assessed by NIHSS and GCS)	Predictive value of SIRI	3	5
Zhang et al.	2020	China	Retrospective cohort study	178	34.83	57.64 ± 10.23	aSAH	Coiling or Clipping	4.105	Functional outcome (assessed by GOS)	Predictive value of SIRI	90 d	7
Zhang et al.	2020	China	Retrospective cohort study	125	36	56.00 ± 12.00	aSAH	Coiling or Clipping	3.63	Vasospasm	Predictive value of SIRI	–	5
Lattanzi et al.	2021	Italy	Retrospective cohort study	184	47.28	75 (61–81)	AIS	EVT	3.8	Futile Recanalization (assessed by mRS)	Predictive value of SIRI	90 d	8
Li et al.	2021	China	Retrospective study based on a prospectively collected database	403 in original cohort 262 in PSM cohort	68.48 in original cohort 67.55 in PSM cohort	58.56 ± 13.28 in original cohort 58.77 ± 13.56 in PSM cohort	ICH	Surgical intervention or conservative treatment	2.76	Functional outcomes (assessed by mRS) mortality	Predictive value of SIRI	90 d for functional outcomes 30 d for mortality	8
Shi et al.	2021	China	Retrospective cohort study	135	40.74	57.03 ± 2.15	aSAH	–	5.91	Poor outcome (assessed by GOS)	HHS, mFS, DCI, Pneumonia, AHC; Predictive value of SIRI	90 d	8
Yi et al.	2021	Korea	Retrospective study based on a prospectively collected database	440	59.09	70.34 ± 12.87	AIS	MT	2.9	Functional outcome (assessed by mRS)	Predictive value of SIRI	90 d	8
Yun et al.	2021	Korea	Retrospective multi-center	680	68.24	56.43 ± 12.96	aSAH	Surgery or Intervention	3.2	Functional outcome (assessed by mRS)	HHS, mFS, DCI, Vasospasm, AHC	90 d	8
Zhang et al.	2021	China	Retrospective cohort	2450 (MIMIC-III database)	52.29	68.17 ± 15.68	Stroke	–	3.8	Mortality	Predictive value of SIRI	In-hospital, 30 d, 90 d, 1 year	7
Li et al.	2022	China	Retrospective single-center	303	58.75	69 (60–78)	AIS	IVT	–	Functional outcome (assessed by mRS)	Predictive value of SIRI	90 d	8
Ma et al.	2022	China	Prospective single-center	63	68.84	65.35 ± 11.33	AIS	IVT with alteplase	1.01	Functional outcome (assessed by mRS)	Severity of AIS (NIHSS) Predictive value of SIRI	90 d	9
Wang et al.	2022	China	Retrospective single-center cohort study	375	65.87	61.9 ± 11.3	AIS	–	0.767	END (assessed by NIHSS)	–	every day for 7 days after admission	7
Yu et al.	2022	China	Retrospective single-center	111	66.67	58.49 ± 15.01	SAH	–	6.478	Functional outcome (assessed by mRS)	HHS, mFS, WFNS, DCI, Vasospasm, AHC, Rebleeding; Predictive value of SIRI	90 d	8
Zhou et al.	2022	China	Prospective single-center	287	68.99	61.55 ± 13.08	AIS	–	1.349	Functional outcome (assessed by mRS)	Predictive value of SIRI	90 d	9
Dang et al.	2023	China	Retrospective cohort	2043 in original cohort 888 in PSM cohort (MIMIC-IV database)	50.37 in original cohort 51.16 in PSM cohort	70.4 (57.9–81.8) in original cohort 71.5 (58.6–82.8) in PSM cohort	AIS	Thrombolysis or thrombectomy	4.57	LOS ICU, LOS hospital, 90 d all-cause mortality	30 d all-cause mortality 1-year all-cause mortality	In-hospital, 30 d, 90 d, 1-year	7
Huang et al.	2023	China	Retrospective single-center cross-sectional study	234	50.43	69 (57–78)	AIS	Thrombolysis or antiplatelet or anticoagulation	1.79	Functional outcome (assessed by mRS)	Severity of AIS (NIHSS) Predictive value of SIRI	–	7
Wang JJ et al.	2023	China	Prospective single-center	640	71.41	56.0 (48.0–64.0)	ICH	–	5.03	Functional outcome (assessed by mRS)	Complicated infections mortality	30 d, 90 d, 1-year	8
Yan et al.	2023	China	Retrospective single-center	2802	61.67	68.02 ± 11.53	AIS	–	2.74	SAP	Predictive value of SIRI	7 days after admission	6
Yu et al.	2023	China	Prospective single-center observational study	378	66.93	63.54 ± 13.72	ICH	–	–	SAP	Predictive value of SIRI	–	7
Chu et al.	2023	China	Retrospective single-center	240	33.75	66.00 (60.00–73.35)	AIS	IVT	1.00	Functional outcome (assessed by mRS)	Predictive value of SIRI	90 d	8
Hou et al.	2023	China	Retrospective cohort	394 in original cohort 200 in PSM cohort	44.87	55.6 ± 9.8	aSAH	Coiling or Clipping	5.36	Functional outcome (assessed by mRS)	Predictive value of SIRI	90 d	9
Wang RH et al.	2023	China	Prospective multi-center observational	320	66.56	62.5 (51.3–73)	ICH	Nasogastric tube surgery	2.291	SAP	Predictive value of SIRI	7 days after admission	9

### Functional outcomes assessed by the GOS or mRS

**Fig. 2:**
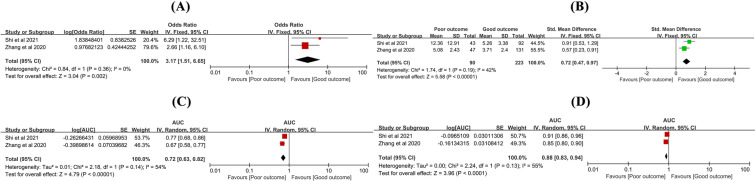
The relationship of SIRI and functional outcomes (assessed by GOS). **A** SIRI for predicting poor outcome; **B** The difference of SIRI values between poor outcome and good outcome; **C** The predictive value of SIRI for poor outcome; **D** The predictive value of SIRI combined with clinical data for poor outcome

Two studies [15, 38] reported functional outcomes assessed by GOS. The meta-analysis showed that individuals with high SIRI had a 3.17-fold higher risk of poor outcomes compared to those with low SIRI (odds ratio [OR] 3.17, 95% confidence interval [CI] 1.51–6.65, *P* = 0.002, *I*^2^ = 0%, Fig. 2A), and the SIRI value was 0.72 higher in those with poor outcomes compared to those with good outcomes (standard mean difference [SMD] 0.72, 95%CI 0.47–0.97, *P* < 0.00001, *I*^2^ = 42%, Fig. 2B). The predictive value of SIRI for poor outcome was 0.72 with a 95%CI of 0.63 to 0.82, *P* < 0.00001, and *I*^2^ = 54% (Fig. 2C). After combining with clinical data, the predictive value for poor outcome was 0.88 with a 95%CI of 0.83 to 0.94, *P* < 0.0001, and *I*^2^ = 55% (Fig. 2D), indicating that SIRI had a reasonably good predictive accuracy and a potential predictive ability. The results are summarized in Table 2.

**Table 2:** Meta-analysis of different outcomes

Items	Results		
	Studies, *n*	OR (95% CI)	*P*-value (Heterogeneity, *I*^2^ and *P* for Cochran *Q*)
Functional outcomes (assessed by GOS)	2	3.17 (1.51–6.65)	*P* = 0.002 (*I*^2^ = 0%, *P* = 0.36)
Functional outcomes (assessed by mRS)			
Continuous variable	7	1.20 (1.07–1.34)	*P* = 0.001 (*I*^2^ = 66%, *P* = 0.008)
Dichotomous variable	7	3.01 (2.00–4.54)	*P* < 0.0001 (*I*^2^ = 74%, *P* = 0.0007)
Mortality			
In-hospital	3	1.68 (1.43–1.97)	*P* < 0.00001 (*I*^2^ = 0%, *P* = 0.37)
1 month	4	1.50 (1.14–1.98)	*P* = 0.004 (*I*^2^ = 85%, *P* = 0.0002)
3 months	3	1.77 (1.53–2.04)	*P* < 0.00001 (*I*^2^ = 0%, *P* = 0.77)
1 year	2	1.65 (1.43–1.92)	*P* < 0.00001 (*I*^2^ = 1%, *P* = 0.31)
SAP			
Continuous variable	3	1.11 (1.05–1.18)	*P* = 0.0006 (*I*^2^ = 66%, *P* = 0.05)
Dichotomous variable	2	2.89 (2.23–3.75)	*P* < 0.00001 (*I*^2^ = 0%, *P* = 0.60)
END	2	1.78 (0.95–3.34)	*P* = 0.07 (*I*^2^ = 85%, *P* = 0.01)
SAH-related clinical parameters			
HHS	4	2.70 (1.45–5.01)	*P* = 0.002 (*I*^2^ = 67%, *P* = 0.03)
mFS	4	2.99 (1.57–5.70)	*P* = 0.0009 (*I*^2^ = 77%, *P* = 0.005)
DCI	3	3.09 (2.16–4.43)	*P* < 0.00001 (*I*^2^ = 0%, *P* = 0.89)
Vasospasm	3	1.67 (1.28–2.17)	*P* = 0.0001 (*I*^2^ = 79%, *P* = 0.008)
AHC	4	1.90 (0.84–4.29)	*P* = 0.12 (*I*^2^ = 81%, *P* = 0.001)
Predictive value of SIRI for poor outcome (assessed by GOS)			
SIRI	2	0.72 (0.63–0.82)	*P* < 0.00001 (*I*^2^ = 54%, *P* = 0.14)
SIRI combining with clinical data	2	0.88 (0.83–0.94)	*P* < 0.0001 (*I*^2^ = 55%, *P* = 0.13)
Predictive value of SIRI for poor outcome (assessed by mRS)	12	0.72 (0.69–0.76)	*P* < 0.00001 (*I*^2^ = 78%, *P* < 0.00001)
Predictive value of SIRI for SAP	4	0.81 (0.74–0.89)	*P* < 0.00001 (*I*^2^ = 90%, *P* < 0.00001)

Items	Results		
	Studies, *n*	SMD or MD (95% CI)	*P* value heterogeneity (*I*^2^, *P* for Cochran *Q*)
SIRI values between different endpoints			
Poor/good outcome (assessed by GOS)	2	0.72 (0.47–0.97)	*P* < 0.00001 (*I*^2^ = 42%, *P* = 0.19)
Poor/good outcome (assessed by mRS)	8	0.61 (0.52–0.69)	*P* < 0.00001 (*I*^2^ = 60%, *P* = 0.01)
SAP and Non-SAP	3	3.24 (1.56–4.91)	*P* = 0.0002 (*I*^2^ = 88%, *P* = 0.0002)
END and Non-END	2	0.37 (0.34–0.40)	*P* < 0.00001 (*I*^2^ = 0%, *P* = 0.79)

**Fig. 3:**
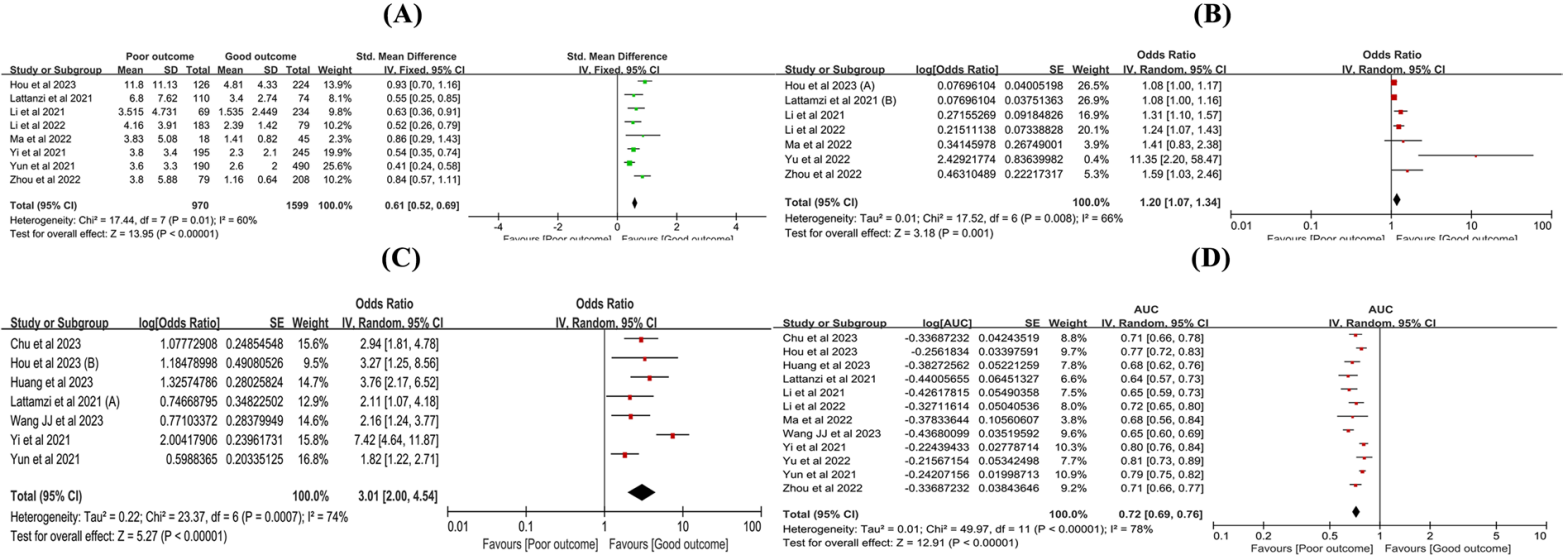
The relationship of SIRI and functional outcomes (assessed by mRS). **A** The difference of SIRI values between poor outcome and good outcome; **B** SIRI for predicting poor outcome (continuous); **C** SIRI for predicting poor outcome (dichotomous); **D** The predictive value of SIRI for poor outcome

Eight studies [9, 14, 16, 24, 25, 29, 39, 41] reported the SIRI values between good and poor outcome group, and the SIRI values were found to be 0.61 higher than that in good outcome with a 95% CI of 0.52 to 0.69, *P* < 0.00001, and *I*^2^ = 60% (Fig. 3A). 12 studies [9, 14, 16, 24, 25, 27–29, 31, 34, 39, 41] assessed functional outcomes using the mRS scale and reported the ORs and 95% CIs for SIRI and poor outcome, with 2 studies [24, 41] considering SIRI as both a continuous and dichotomous variable. The meta-analysis of 7 studies [9, 24, 25, 27, 29, 39, 41] considering SIRI as a continuous variable showed that for each standard deviation increase in SIRI, the risk of poor outcome increased by 20% (OR 1.20, 95% CI 1.07–1.34, *P* = 0.001, *I*^2^ = 66%, Fig. 3B). The meta-analysis of 7 studies [14, 16, 24, 28, 31, 34, 41] considering SIRI as a dichotomous variable showed that high SIRI was associated with a higher risk of poor outcome compared to low SIRI (OR 3.01, 95% CI 2.00–4.54, *P* < 0.0001, *I*^2^ = 74%, Fig. 3C). The predictive value of SIRI for poor outcome was 0.72 with a 95% CI 0.69 to 0.76, *P* < 0.00001, and *I*^2^ = 78% (Fig. 3D). The results are summarized in Table 2.

In summary, despite the use of different assessment tools for poor outcome, it was consistently found that high SIRI was strongly associated with poor outcomes. In other words, there was a significant correlation between high SIRI and poor outcome.

### SIRI and mortality

**Fig. 4:**
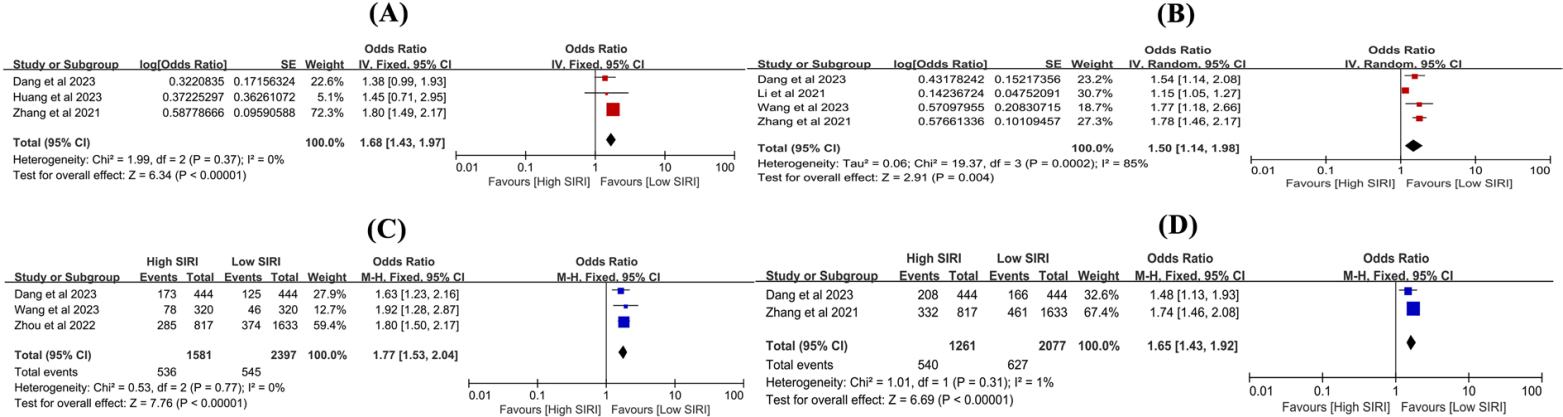
The relationship of SIRI and mortality. **A** SIRI for predicting in-hospital mortality; **B** SIRI for predicting 1-month mortality; **C** SIRI for predicting 3-month mortality; **D** SIRI for predicting 1 year mortality

Four studies [9, 10, 30, 31] reported mortality rates ranging from in-hospital to 1 year after discharge. The meta-analysis showed that a high SIRI was associated with a 1.68-fold increased risk for in-hospital mortality (OR 1.68, 95% CI 1.43–1.97, *P* < 0.00001, *I*^2^ = 0%, Fig. 4A), a 1.50-fold increased risk for 1-month mortality (OR 1.50, 95% CI 1.14–1.98, *P* = 0.004, *I*^2^ = 85%, Fig. 4B), a 1.77-fold increased risk for 3-month mortality (OR 1.77, 95% CI 1.53–2.04, *P* < 0.00001, *I*^2^ = 0%, Fig. 4C), and a 1.65-fold increased risk for 1-year mortality (OR 1.65, 95% CI 1.43–1.92, *P* < 0.00001, *I*^2^ = 1%, Fig. 4D) when compared to those with low SIRI. The results are summarized in Table 2.

### SIRI and SAP

**Fig. 5:**
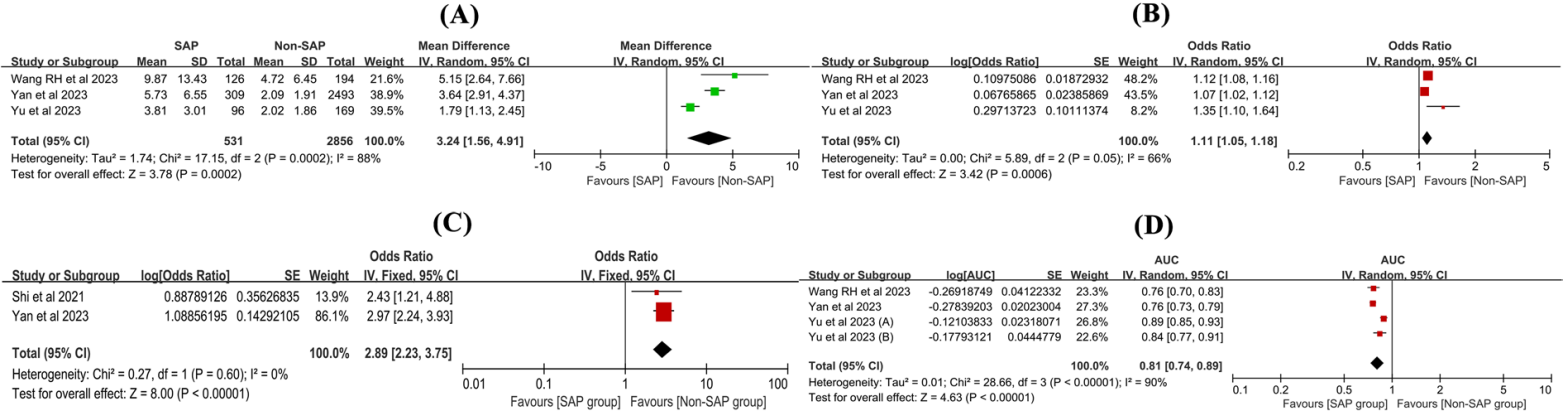
The relationship of SIRI and SAP. **A** The difference of SIRI values between SAP and Non-SAP; **B** SIRI for predicting SAP (continuous); **C** SIRI for predicting SAP (dichotomous); **D** The predictive value of SIRI for SAP

Three studies [32, 33, 42] reported the SAP. The SIRI value of SAP was increased by 3.24 than non-SAP with 95% CI 1.56 to 4.91, *P* = 0.0002 and *I*^2^ = 88% (Fig. 5A). 4 studies [32, 33, 38, 42] reported the ORs and 95CIs for SAP, in which one study [32] regarded the SIRI values as continuous variable and dichotomous variable. Three studies [32, 33, 42] regarded the SIRI value as continuous variable and the meta-analysis showed that for each standard deviation increase in SIRI, the risk of SAP increased by 11% (OR 1.11, 95% CI 1.05–1.18, *P* = 0.0006, *I*^2^ = 66%, Fig. 5B). Two studies [32, 38] regarded the SIRI value as dichotomous variable and the meta-analysis showed that high SIRI had 2.89-folds risk for SAP comparing low SIRI (OR 2.89, 95% CI 2.23–3.75, *P* < 0.00001, *I*^2^ = 0%, Fig. 5C). One study [33] randomized patients into the training and validation cohorts, and the two cohorts were regarded as two independent studies. The predictive value of SIRI for SAP was 0.81 with 95%CI ranged from 0.74 to 0.89, *P* < 0.00001, *I*^2^ = 90% (Fig. 5D). The results are summarized in Table 2.

### SIRI and END after stroke

**Fig. 6:**
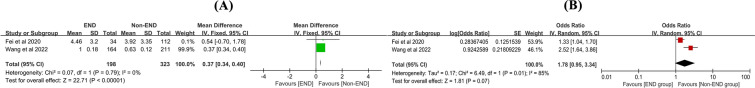
The relationship of SIRI and END. **A** The difference of SIRI values between END and Non-END; **B** SIRI for predicting END

Two studies [26, 36] provided data on END. The SIRI value of END was found to be 0.37 higher than that of non-END with a 95% CI of 0.34 to 0.40, *P* < 0.00001 and *I*^2^ = 0% (Fig. 6A). However, the meta-analysis revealed that high SIRI did not significantly increase the risk of END compared to low SIRI (OR 1.78, 95% CI 0.95–3.34, *P* = 0.07, *I*^2^ = 85%, Fig. 6B). The results are summarized in Table 2.

### SIRI and SAH-related clinical parameters

**Fig. 7:**
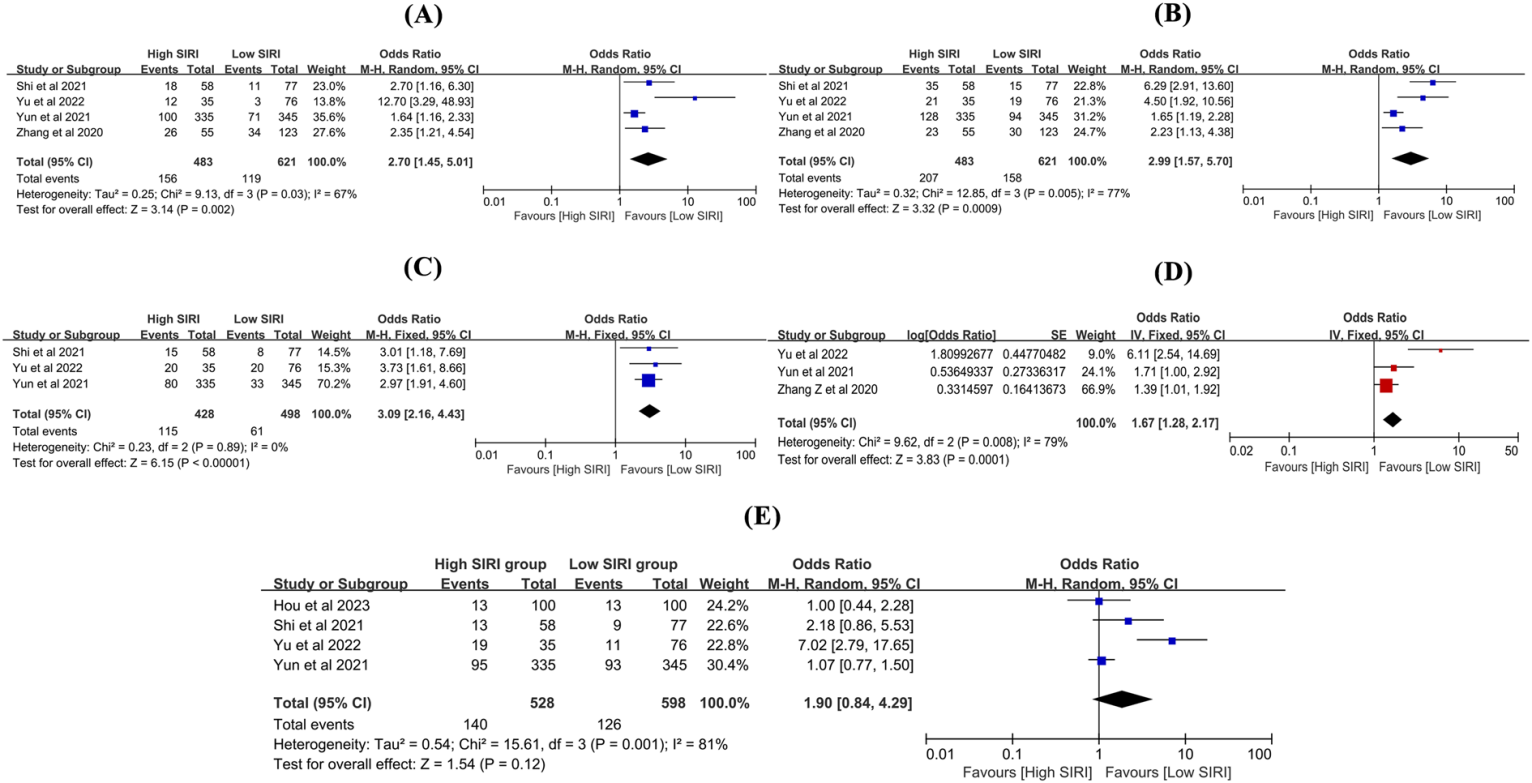
The relationship of SIRI and SAH-associated clinical parameters. The **A** HHS, **B** mFS, **C** DCI, **D** vasospasm, and **E** AHC between high SIRI and low SIRI

Five studies [14, 15, 27, 38, 41] investigated the association between SIRI and SAH-related clinical parameters. The meta-analysis indicated that high SIRI was usually associated with higher scores for HHS (OR 2.70, 95% CI 1.45–5.01, *P* = 0.002, *I*^2^ = 67%, Fig. 7A), mFS (OR 2.99, 95% CI 1.57–5.70, *P* = 0.0009, *I*^2^ = 77%, Fig. 7B), increased risk of DCI (OR 3.09, 95% CI 2.16–4.43, *P* < 0.00001, *I*^2^ = 0%, Fig. 7C), and vasospasm (OR 1.67, 95% CI 1.28–2.17, *P* = 0.0001, *I*^2^ = 79%, Fig. 7D) compared to low SIRI. However, the risk of AHC (OR 1.90, 95% CI 0.84–4.29, *P* = 0.12, *I*^2^ = 81%, Fig. 7E) was not statistically significant between the two groups. It is noteworthy that HHS, mFS, DCI, vasospasm, and AHC are all indicators of SAH severity, indicating that high SIRI was associated with more severe SAH. In regions with limited medical resources and where CT scans are not readily available, this simple index may prove valuable in predicting SAH severity and patient stratification. The results are summarized in Table 2.

### Analyses of subgroups based on sub-type of stroke

**Fig. 8:**
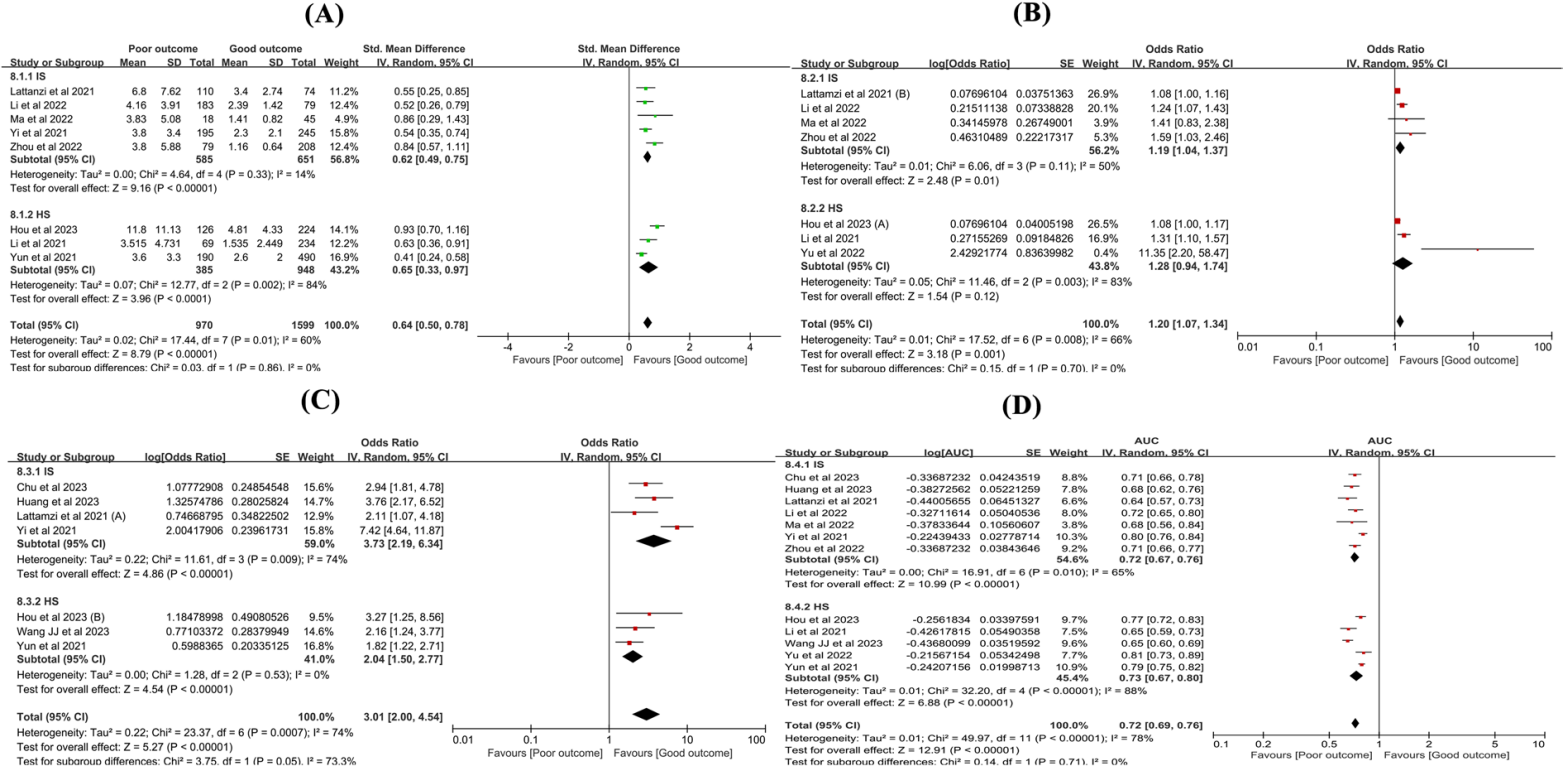
Subgroup analyses based on sub-type of stroke. **A** The difference of SIRI values between poor outcome and good outcome; **B** SIRI for predicting poor outcome (continuous); **C** SIRI for predicting poor outcome (dichotomous); **D** The predictive value of SIRI for poor outcome

Subgroup analyses were conducted based on the sub-type of stroke (IS and HS) for (i) the difference in SIRI values between the poor outcome group and the good outcome group, (ii) predicting poor outcome when SIRI was regarded as a continuous variable or dichotomous variable, and (iii) the predictive value of SIRI for poor outcome. Subgroup analysis demonstrated that the SIRI values were higher in the poor outcome group than in the good outcome group for both IS (SMD: 0.62; 95% CI 0.49–0.75, *P* < 0.00001, *I*^2^ = 14%) and HS (SMD: 0.65; 95% CI 0.33–0.67, *P* < 0.00001, *I*^2^ = 84%) (Fig. 8A). When SIRI was regarded as a continuous variable, subgroup analysis demonstrated that for each standard deviation increase in SIRI, the risk of poor outcome increased by 19% for IS (OR: 1.19; 95% CI 1.04–1.37, *P* = 0.01, and *I*^2^ = 50%), whereas no statistically significant difference was found for HS (OR: 1.28; 95% CI 0.94–1.74, *P* = 0.12, and *I*^2^ = 83%) (Fig. 8B). Similarly, when SIRI was regarded as a dichotomous variable, subgroup analysis demonstrated that the risk of a poor outcome at a high SIRI level was 3.73 times greater than that at a low SIRI level for IS (OR: 3.73; 95% CI 2.19–6.34, *P* < 0.00001, and *I*^2^ = 74%) and 2.04 times greater for HS (OR: 2.04; 95% CI 1.50–2.77, *P* < 0.00001, *I*^2^ = 0%) (Fig. 8C). Lastly, the predictive value of SIRI for poor outcomes was 0.72 for IS (AUC: 0.72; 95% CI 0.67–0.76) and 0.73 for HS (AUC: 0.73; 95% CI 0.67–0.80) (Fig. 8D). The results are summarized in Table 3.

**Table 3:** Subgroup analyses based on sub-type of stroke

Items	Results		
	studies, *n*	SMD/OR/AUC (95% CI)	*P*-value (heterogeneity, *I*^2^ and *P* for Cochran *Q)*
SIRI values between poor and good outcome^§^			
IS	5	0.62 (0.49–0.75)	*P* < 0.00001 (*I*^2^ = 14%, *P* = 0.33)
HS	3	0.65 (0.33–0.97)	*P* < 0.00001 (*I*^2^ = 60%, *P* = 0.002)
Poor outcome (continuous variable)^*^			
IS	4	1.19 (1.04–1.37)	*P* = 0.01 (*I*^2^ = 50%, *P* = 0.11)
HS	3	1.28 (0.94–1.74)	*P* = 0.12 (*I*^2^ = 83%, *P* = 0.003)
Poor outcome (dichotomous variable)^*^			
IS	4	3.73 (2.19–6.43)	*P* < 0.00001 (*I*^2^ = 74%, *P* = 0.009)
HS	3	2.04 (1.50–2.77)	*P* < 0.00001 (*I*^2^ = 0%, *P* = 0.53)
Predictive value of SIRI for poor outcome^※^			
IS	7	0.72 (0.67–0.76)	*P* < 0.00001 (*I*^2^ = 65%, *P* = 0.010)
HS	5	0.73 (0.67–0.80)	*P* < 0.00001 (*I*^2^ = 88%, *P* < 0.00001)

* represents the data are expressed as OR

§ represents the data are expressed as SMD

※ represents the data are expressed as AUC

### Risk of bias assessment and publication bias assessment

The NOS has assessed and awarded a median of 8 stars to all the research, with an inter-quartile range of 5 to 9 stars. The methodological quality of the studies included can be found in Additional file 1: Table S4. Additionally, the probability of publication bias was evaluated through funnel plot results, which are displayed in Additional file 1: Figure S1.

## Discussion

Secondary brain tissue damage after AIS [43, 44] is attributed to the inflammatory reaction. Inflammatory cells of the immune system secrete different substances, such as cytokines, adhesion molecules, and chemokines, which worsen the harm to tissues. Earlier research has indicated that the inflammatory reaction can be promptly initiated following a stroke and is closely associated with unfavorable consequences [45–47]. The investigation of biomarkers is focused on various inflammatory factors linked to stroke, which are emphasized by these mechanisms.

The importance of inflammation in the development of stroke has been confirmed by many research studies. In every step of atherosclerotic plaque development, inflammation plays a crucial role and leads to the occurrence of thrombotic events [48]. The beginning of early plaque formation is marked by monocyte attachment to the vascular endothelium, movement into the arterial intima, and later transformation into foamy macrophages [49, 50]. The occurrence of stroke is frequently a result of the disturbance of atherosclerotic plaques, which is linked to the infiltration of monocyte/macrophage and T-cells [51]. Furthermore, inflammation is crucial in the pathophysiological processes of brain damage. After ischemia, white blood cells escape from the bloodstream and enter the brain and meninges [52]. The brain is harmed by neutrophils when they release enzymes like metalloproteases (MMP-9), cathepsin G, reactive oxygen and nitrogen compounds, and the inflammatory IL-1β [53]. The arrival of monocyte-derived macrophages (MDMs) in the ischemic brain may play a vital role in controlling the immune reaction following a stroke [54, 55]. Additionally, stroke can activate systemic inflammation and neurohumoral pathways, leading to immune activation, immunodepression, and functional impairment of various peripheral organs [53, 55–59]. Therefore, markers of inflammation might suggest the prognosis after a stroke.

The SIRI is an innovative and comprehensive indicator that relies on the absolute values of neutrophil, monocyte, and lymphocyte counts in the peripheral blood. During the initiation of stroke, the activation of peripheral circulating neutrophils occurs first, leading to the release of antimicrobial enzymes and chemical substances that worsen brain damage [60, 61]. In the initial phase of AIS, elevated neutrophil counts were linked to greater infarction size, suggesting that the rise in neutrophil levels may worsen blood–brain barrier damage by facilitating excessive matrix metalloproteinase-9 expression [62, 63]. Furthermore, following AIS, monocytes serve as another crucial category of inflammatory cells capable of infiltrating infarct locations and exacerbating cerebral harm [64–66]. Contrary to neutrophils and monocytes, certain lymphocytes have a crucial function in controlling and diminishing local inflammation during the inflammatory response after AIS, thereby providing protection [67]. Hence, a substantial SIRI (N↑ × M↑/L↓) can precisely indicate the adaptive immune response and inflammation response, which play a crucial role in the occurrence of stroke and hold potential as a reliable prognostic indicator. Furthermore, these three types of blood cells symbolize distinct pathways related to inflammation and immunity within the body, thereby offering a more holistic indication of the body’s inflammatory condition.

Previous studies have demonstrated that the SIRI is an effective marker for assessing the clinical prognosis of various stroke types, including AIS, ICH, and SAH. Fei et al. [36] have shown that SIRI is closely correlated with the occurrence of END in basal ganglia ICH patients and has predictive value in improving the early identification and screening of END and patient outcomes. Wang et al. [26] have reported that SIRI can serve as a new predictor of END in a more objective and reliable manner, as well as a monitor of treatment response. However, our analysis indicates that high SIRI does not increase the risk of END compared with low SIRI. As only 2 studies have focused on the relationship between SIRI and END after stroke, further research is necessary and urgent. In another study, Lin et al. [35] investigated the association between SIRI and atrial fibrillation and found that elevated SIRI values are potential biomarkers of AF among IS patients. However, as there is limited research on the relationship between SIRI and cardiovascular disease, further exploration is warranted. Yu et al. [33] studied the relationship between SIRI and SAP and demonstrated that SIRI at admission can be used as a prognostic inflammatory biomarker in ICH patients with SAP. Yan et al. [32] also reported that SIRI has a good predictive value for SAP, and stroke patients with high SIRI levels (≥ 2.74) should be aware of the risk of SAP. Our analysis showed that although there was no dose–response relationship between SIRI and SAP, high SIRI had a 2.89-fold risk for SAP compared with low SIRI.

As we are aware, SIRI has emerged as a promising prognostic indicator for stroke patients. However, it is essential to consider potential confounding factors that may affect SIRI values, such as infections that develop or coexist with stroke, especially in the elderly population who are susceptible to aspiration pneumonia and urine infections. Moreover, the ongoing COVID-19 pandemic has further complicated the situation, as almost all stroke patients have a compromised and diminished immune system, which could interfere with blood cell count and, consequently, affect SIRI values. Therefore, it is imperative to accurately document comorbidities, including infections and COVID-19 infection status, and pay closer attention to the basic conditions of elderly patients to make appropriate adjustments in data analysis. Future investigations should also consider the influence of stroke patients’ histories of infection to obtain a more comprehensive understanding of SIRI as a prognostic marker for stroke outcomes. Overall, a more in-depth investigation into the relationship between SIRI, infection, and stroke outcomes, taking into account potential confounding factors, could provide more valuable insights for improving stroke management and patient outcomes.

To our knowledge, this is the first systematic review and meta-analysis to investigate the association between SIRI and clinical outcomes in stroke patients. Our analysis demonstrated that high SIRI values were associated with poor outcomes regardless of the assessment tools used. Furthermore, high SIRI values were related to both short-term and long-term mortality and could indicate the severity of SAH. In other words, higher SIRI values indicated more severe SAH. In places where CT scans are not available and medical conditions are poor, this simple index may play an important role in predicting the severity of SAH and stratifying patients. The predictive value of SIRI for poor outcomes and SAP was relatively high, with adverse endpoints typically having higher SIRI values.

### Limitations

While our study provides important insights into the association between SIRI and stroke patient outcomes, it is important to acknowledge several limitations. Firstly, due to the nature of inflammation response in stroke, most of the existing literature on this topic comprises retrospective studies, which may introduce limitations in terms of sample size, confounding variables, and selection bias. Secondly, with the exception of four prospective studies, the majority of studies included in our analysis were retrospective, resulting in considerable heterogeneity in data reporting and follow-up protocols. Therefore, further high-quality prospective studies are needed to confirm the validity and generalizability of our findings. Thirdly, based on our systematic review, the majority of included studies (86%, 19 out of 22 studies) were carried out in China, with two studies from the MIMIC database. As we know, the MIMIC database was established by the Beth Israel Deaconess Medical Center (Boston, MA, USA), and the population consisted mainly of US citizens. Therefore, these two studies reflected the relationship between SIRI and clinical outcomes in Americans. But the existing literature still lacks related studies in Europe or Africa. The broader applicability of SIRI as a predictive tool for stroke outcomes should be identified further in other ethnicities and countries. Fourthly, the high heterogeneity observed in some of our endpoints could influence the robustness of our results. Fifthly, some results are not mirrored to the total population of our studies selected, for each variable evaluated a different lesser number of studies were included. Hence, some findings are less robust. Despite these limitations, our meta-analysis provides valuable preliminary findings that could assist clinicians in making informed treatment decisions for stroke patients. Future research should aim to address these limitations and provide further insights into the association between SIRI and stroke outcomes.

## Conclusion

This study could potentially pave the way for groundbreaking insights into the relationship between SIRI and stroke patient outcomes, as it appears to be the first meta-analysis to explore this association. Given the critical role of the inflammatory response in stroke recovery, closely monitoring patients with high SIRI levels could represent a promising strategy for mitigating brain damage post-stroke. Thus, further investigation into SIRI and its impact on clinical outcomes is essential. While our initial findings offer valuable insights into this area, continued research is necessary to fully elucidate the potential of SIRI, ideally through dynamic monitoring and large-scale, multi-center studies. Ultimately, this research has the potential to inform clinical decision-making and improve patient outcomes following stroke.

### Supplementary Information

**Additional file 1: Table S1.** PRISMA 2020 checklist. **Table S2.** Search strategy. **Table S3.** The definition of mRS and GOS. **Table S4.** ROB assessment for the quality of studies in meta-analysis. **Figure S1.** Funnel plot results of main end points.

## Data Availability

The original contributions presented in the study are included in the article/Additional Material. Any additional queries regarding the research should be directed towards the corresponding author. The additional material for this article can be found online.

## References

[1] Claiborne Johnston S, Mendis S, Mathers CD (2009). Global variation in stroke burden and mortality: estimates from monitoring, surveillance, and modelling. Lancet Neurol.

[2] Zhao D, Liu J, Wang M (2018). Epidemiology of cardiovascular disease in China: current features and implications. Nat Rev Cardiol.

[3] Randolph SA (2016). Ischemic stroke. Workplace Health Saf.

[4] Collaborators GS (2021). Global, regional, and national burden of stroke and its risk factors, 1990–2019: a systematic analysis for the Global Burden of Disease Study 2019. Lancet Neurol.

[5] Suarez JI, Tarr RW, Selman WR (2006). Aneurysmal subarachnoid hemorrhage. N Engl J Med.

[6] Feigin VL, Norrving B, Mensah GA (2017). Global burden of stroke. Circ Res.

[7] O’Carroll CB, Brown BL, Freeman WD (2021). Intracerebral hemorrhage: a common yet disproportionately deadly stroke subtype. Mayo Clin Proc.

[8] Wang YJ, Li ZX, Gu HQ (2022). China stroke statistics: an update on the 2019 report from the National Center for Healthcare Quality Management in Neurological Diseases, China National Clinical Research Center for Neurological Diseases, the Chinese Stroke Association, National Center for Chronic and Non-communicable Disease Control and Prevention, Chinese Center for Disease Control and Prevention and Institute for Global Neuroscience and Stroke Collaborations. Stroke Vasc Neurol.

[9] Li J, Yuan Y, Liao X (2021). Prognostic significance of admission systemic inflammation response index in patients with spontaneous intracerebral hemorrhage: a propensity score matching analysis. Front Neurol.

[10] Zhang Y, Xing Z, Zhou K (2021). The predictive role of systemic inflammation response index (SIRI) in the prognosis of stroke patients. Clin Interv Aging.

[11] Qi Q, Zhuang L, Shen Y, Geng Y, Yu S, Chen H (2016). A novel systemic inflammation response index (SIRI) for predicting the survival of patients with pancreatic cancer after chemotherapy. Cancer.

[12] Li S, Xu H, Wang W, Gao H, Li H, Zhang S (2019). The systemic inflammation response index predicts survival and recurrence in patients with respectable pancreatic ductal adenocarcinoma. Cancer Manag Res.

[13] Wei L, Xie H, Yan P (2020). Prognostic value of the systemic inflammation response index in human malignancy: a meta-analysis. Medicine (Baltimore).

[14] Yun S, Yi HJ, Lee DH (2021). Systemic inflammation response index and systemic immune-inflammation index for predicting the prognosis of patients with aneurysmal subarachnoid hemorrhage. J Stroke Cerebrovasc Dis.

[15] Zhang P, Li Y, Zhang H (2020). Prognostic value of the systemic inflammation response index in patients with aneurismal subarachnoid hemorrhage and a Nomogram model construction. Br J Neurosurg.

[16] Yi HJ, Sung JH, Lee DH (2021). Systemic Inflammation response index and systemic immune-inflammation index are associated with clinical outcomes in patients treated with mechanical thrombectomy for large artery occlusion. World Neurosurg.

[17] Jin Z, Hao D, Song Y (2021). Systemic inflammatory response index as an independent risk factor for ischemic stroke in patients with rheumatoid arthritis: a retrospective study based on propensity score matching. Clin Rheumatol.

[18] Page MJ, McKenzie JE, Bossuyt PM (2021). The PRISMA 2020 statement: an updated guideline for reporting systematic reviews. J Clin Epidemiol.

[19] Huang YW, Zhang Y, Feng C, An YH, Li ZP, Yin XS. Systemic inflammation response index as a clinical outcome evaluating tool and prognostic indicator for hospitalized stroke patients: a systematic review and meta-analysis. PROSPERO. 2023; CRD42023405221. https://www.crd.york.ac.uk/prospero/display_record.php?ID$=$CRD42023405221. Accessed 25 March 2023. 10.1186/s40001-023-01446-3 PMC10621190 37915088

[20] Wells GA, Shea B, O’Connell D, Peterson J, Welch V, Losos M, et al. The Newcastle-Ottawa Scale (NOS) for assessing the quality of nonrandomized studies in meta-analyses. http://www.ohri.ca/programs/clinical_epidemiology/oxford.htm. Accessed 27 Feb 2020.

[21] McGrath S, Zhao X, Steele R, Thombs BD, Benedetti A, the DEPRESsion Screening Data (DEPRESSD) Collaboration (2020). Estimating the sample mean and standard deviation from commonly reported quantiles in meta-analysis. Stat Methods Med Res.

[22] DerSimonian R, Laird N (1986). Meta-analysis in clinical trials. Control Clin Trials.

[23] Higgins JP, Thompson SG (2002). Quantifying heterogeneity in a meta-analysis. Stat Med.

[24] Lattanzi S, Norata D, Divani AA, Di Napoli M, Broggi S, Rocchi C (2021). Systemic inflammatory response index and futile recanalization in patients with ischemic stroke undergoing endovascular treatment. Brain Sci.

[25] Ma X, Yang J, Wang X, Wang X, Chai S (2022). The clinical value of systemic inflammatory response index and inflammatory prognosis index in predicting 3-month outcome in acute ischemic stroke patients with intravenous thrombolysis. Int J Gen Med.

[26] Wang J, Zhang X, Tian J, Li H, Tang H, Yang C (2022). Predictive values of systemic inflammatory responses index in early neurological deterioration in patients with acute ischemic stroke. J Integr Neurosci.

[27] Yu TT, Wang ZL (2022). Use of a systemic inflammatory response index to predict non-traumatic non-aneurysmal subarachnoid hemorrhage patient outcomes. J Stroke Cerebrovasc Dis.

[28] Huang L (2023). Increased systemic immune-inflammation index predicts disease severity and functional outcome in acute ischemic stroke patients. Neurologist.

[29] Zhou Y, Zhang Y, Cui M, Zhang Y, Shang X (2022). Prognostic value of the systemic inflammation response index in patients with acute ischemic stroke. Brain Behav.

[30] Dang H, Mao W, Wang S, Sha J, Lu M, Cong L (2023). Systemic inflammation response index as a prognostic predictor in patients with acute ischemic stroke: a propensity score matching analysis. Front Neurol.

[31] Wang J, Du Y, Wang A, Zhang X, Bian L, Lu J, Zhao X, Wang W (2023). Systemic inflammation and immune index predicting outcomes in patients with intracerebral hemorrhage. Neurol Sci.

[32] Yan D, Dai C, Xu R, Huang Q, Ren W (2023). Predictive ability of systemic inflammation response index for the risk of pneumonia in patients with acute ischemic stroke. Gerontology.

[33] Yu T, Liu H, Liu Y, Jiang J (2023). Inflammatory response biomarkers nomogram for predicting pneumonia in patients with spontaneous intracerebral hemorrhage. Front Neurol.

[34] Chu M, Luo Y, Wang D, Liu Y, Wang D, Wang Y, Zhao J (2023). Systemic inflammation response index predicts 3-month outcome in patients with mild acute ischemic stroke receiving intravenous thrombolysis. Front Neurol.

[35] Lin KB, Fan FH, Cai MQ, Yu Y, Fu CL, Ding LY (2022). Systemic immune inflammation index and system inflammation response index are potential biomarkers of atrial fibrillation among the patients presenting with ischemic stroke. Eur J Med Res.

[36] Fei XB, Zhou XM, Xue XC, Hong K, Gao H (2020). Relationship between siri and early neurological deterioration in basal ganglia cerebral hemorrhage and construction of nomogram predictive model. Int J Surg.

[37] Zhang Z, Zhang HZ, Li YP, Yan ZC, Dong L, Wang XD (2020). Relationship between systemic inflammation response index and symptomatic cerebral vasospasm after aneurismal subarachnoid hemorrhage as well as construction of a Nomogram predictive model. J Clin Med Pract.

[38] Shi XY, Peng HP (2021). Predictive effect of systemic inflammation response index combined with blood glucose/blood potassium ratio on poor prognosis of patients with aneurysmal subarachnoid hemorrhage. Chin For Med Res.

[39] Li LL, Chen ZB, Lin YJ, Cao J, Chen XL (2022). Systemic inflammatory response index predicts outcomes after intravenous thrombolysis in patients with acute ischemic stroke. Int J Cerbrovasc Dis.

[40] Zhang P, Li YP, Wang XD, Tang C, Zhu L, Wan ZQ (2020). Value of nomogram model combined with inflammatory response index in predicting prognosis of aSAH patients. J Clin Neurosurg.

[41] Hou Y, Fan J, Yuan H, Zheng H, Yang H, Li H (2023). Prognostic capacity of the systemic inflammation response index for functional outcome in patients with aneurysmal subarachnoid hemorrhage. Front Neurol.

[42] Wang RH, Wen WX, Jiang ZP, Du ZP, Ma ZH, Lu AL (2023). The clinical value of neutrophil-to-lymphocyte ratio (NLR), systemic immune-inflammation index (SII), platelet-to-lymphocyte ratio (PLR) and systemic inflammation response index (SIRI) for predicting the occurrence and severity of pneumonia in patients with intracerebral hemorrhage. Front Immunol.

[43] Wu F, Liu Z, Zhou L, Ye D, Zhu Y, Huang K (2022). Systemic immune responses after ischemic stroke: from the center to the periphery. Front Immunol.

[44] Sadeghi F, Sarkady F, Zsóri K, Szegedi I, Orbán-Kálmándi R, Székely E (2022). High neutrophil-lymphocyte ratio and low lymphocyte-monocyte ratio combination after thrombolysis is a potential predictor of poor functional outcome of acute ischemic stroke. J Personal Med.

[45] Feng Y, Bai X, Li W, Cao W, Xu X, Yu F (2022). Postoperative neutrophillymphocyte ratio predicts unfavorable outcome of acute ischemic stroke patients who achieve complete reperfusion after thrombectomy. Front Immunol.

[46] Wu F, Wang Q, Qiao Y, Yu Q, Wang F (2022). A new marker of short-term mortality and poor outcome in patients with acute ischemic stroke: mean platelet volume-to lymphocyte ratio. Medicine.

[47] Stuckey S, Ong L, Collins-Praino L, Turner R (2021). Neuroinflammation as a key driver of secondary neurodegeneration following stroke?. Int J Mol Sci.

[48] Kelly PJ, Lemmens R, Tsivgoulis G (2021). Inflammation and stroke risk: a new target for prevention. Stroke.

[49] Soehnlein O, Libby P (2021). Targeting inflammation in atherosclerosis-from experimental insights to the clinic. Nat Rev Drug Discov.

[50] Bäck M, Yurdagul A, Tabas I, Öörni K, Kovanen PT (2019). Inflammation and its resolution in atherosclerosis: mediators and therapeutic opportunities. Nat Rev Cardiol.

[51] Spagnoli LG, Mauriello A, Sangiorgi G, Fratoni S, Bonanno E, Schwartz RS (2004). Extracranial thrombotically active carotid plaque as a risk factor for ischemic stroke. JAMA.

[52] Iadecola C, Buckwalter MS, Anrather J (2020). Immune responses to stroke: mechanisms, modulation, and therapeutic potential. J Clin Invest.

[53] Denorme F, Portier I, Rustad JL, Cody MJ, de Araujo CV, Hoki C (2022). Neutrophil extracellular traps regulate ischemic stroke brain injury. J Clin Invest.

[54] Garcia-Bonilla L, Brea D, Benakis C, Lane DA, Murphy M, Moore J (2018). Endogenous protection from ischemic brain injury by preconditioned monocytes. J Neurosci.

[55] Chauhan A, Al Mamun A, Spiegel G, Harris N, Zhu L, McCullough LD (2018). Splenectomy protects aged mice from injury after experimental stroke. Neurobiol Aging.

[56] Chapman KZ, Dale VQ, Dénes A, Bennett G, Rothwell NJ, Allan SM (2009). A rapid and transient peripheral inflammatory response precedes brain inflammation after experimental stroke. J Cereb Blood Flow Metab.

[57] Xu S, Lu J, Shao A, Zhang JH, Zhang J (2020). Glial cells: role of the immune response in ischemic stroke. Front Immunol.

[58] Tang Y, Xu H, Du X, Lit L, Walker W, Lu A (2006). Gene expression in blood changes rapidly in neutrophils and monocytes after ischemic stroke in humans: a microarray study. J Cereb Blood Flow Metab.

[59] Westendorp WF, Dames C, Nederkoorn PJ, Meisel A (2022). Immunodepression, infections, and functional outcome in ischemic stroke. Stroke.

[60] Kolaczkowska E, Kubes P (2013). Neutrophil recruitment and function in health and inflammation. Nat Rev Immunol.

[61] Jickling GC, Liu D, Ander BP, Stamova B, Zhan X, Sharp FR (2015). Targeting neutrophils in ischemic stroke: translational insights from experimental studies. J Cereb Blood Flow Metab.

[62] Buck BH, Liebeskind DS, Saver JL, Bang OY, Yun SW, Starkman S (2008). Early neutrophilia is associated with volume of ischemic tissue in acute stroke. Stroke.

[63] Garau A, Bertini R, Colotta F, Casilli F, Bigini P, Cagnotto A (2005). Neuroprotection with the Cxcl8 inhibitor repertaxin in transient brain ischemia. Cytokine.

[64] Kaito M, Araya S, Gondo Y, Fujita M, Minato N, Nakanishi M (2013). Relevance of distinct monocyte subsets to clinical course of ischemic stroke patients. PLoS ONE.

[65] Jin R, Yang G, Li G (2010). Inflammatory mechanisms in ischemic stroke: role of inflammatory cells. J Leukoc Biol.

[66] Ray MJ, Walters DL, Bett JN, Cameron J, Wood P, Aroney CN (2005). Platelet-monocyte aggregates predict troponin rise after percutaneous coronary intervention and are inhibited by abciximab. Int J Cardiol.

[67] Liesz A, Zhou W, Na SY, Hämmerling GJ, Garbi N, Karcher S (2013). Boosting regulatory T cells limits neuroinflammation in permanent cortical stroke. J Neurosci.

