# Supplementary material for: Systemic inflammation response index as a clinical outcome evaluating tool and prognostic indicator for hospitalized stroke patients: a systematic review and meta-analysis
Source: Eur J Med Res. 2023 Nov 1;28:474. doi: 10.1186/s40001-023-01446-3 (PMC10621190; doi:10.1186/s40001-023-01446-3)
Supplement: Supplementary file 1 — Additional file 1: Table S1. PRISMA 2020 checklist. Table S2. Search strategy. Table S3. The definition of mRS and GOS. Table S4. ROB assessment for the quality of studies in meta-analysis. Figure S1. Funnel plot results of main end points. [file 40001_2023_1446_MOESM1_ESM.doc]

# Additional Materials

**Additional to:** Yong-Wei Huang, Ye Zhang, Cui Feng, Yin-Hua An , Zong-Ping Li, Xiao-Shuang Yin. Systemic inflammation response index as a clinical outcome evaluating tool and prognostic indicator for hospitalized stroke patients: a systematic review and meta-analysis.

**Contents**

[*Table S1.* ***PRISMA 2020 checklist***](#__RefHeading___Toc49954750).

[*Table S2.* ***Search strategy***](#__RefHeading___Toc49954750).

**Table S3.** The definition of mRS and GOS.

**Table S4.** ROB assessment for the quality of studies in meta-analysis.

**Figure S1.** Funnel plot results of main end points.

[**Table S1.** PRISMA 2020 checklist](#__RefHeading___Toc49954750).

| **Section and Topic** | **Item #** | **Checklist item** | **Location where item is reported** |
| --- | --- | --- | --- |
| **TITLE** | | |  |
| Title | 1 | Identify the report as a systematic review. | page 1 |
| **ABSTRACT** | | |  |
| Abstract | 2 | See the PRISMA 2020 for Abstracts checklist. | page 2 |
| **INTRODUCTION** | | |  |
| Rationale | 3 | Describe the rationale for the review in the context of existing knowledge. | page 3 |
| Objectives | 4 | provide an explicit statement of the objective(s) or question(s) the review addresses. | page 3 |
| **METHODS** | | |  |
| Eligibility criteria | 5 | Specify the inclusion and exclusion criteria for the review and how studies were grouped for the syntheses. | page 3 |
| Information sources | 6 | Specify all databases, registers, websites, organisations, reference lists and other sources searched or consulted to identify studies. Specify the date when each source was last searched or consulted. | page 3 |
| Search strategy | 7 | present the full search strategies for all databases, registers, and websites, including any filters and limits used. | page 3-4 |
| Selection process | 8 | Specify the methods used to decide whether a study met the inclusion criteria of the review, including how many reviewers screened each record and each report retrieved, whether they worked independently, and if applicable, details of automation tools used in the process. | page 4 |
| Data collection process | 9 | Specify the methods used to collect data from reports, including how many reviewers collected data from each report, whether they worked independently, any processes for obtaining or confirming data from study investigators, and if applicable, details of automation tools used in the process. | page 4 |
| Data items | 10a | List and define all outcomes for which data were sought. Specify whether all results that were compatible with each outcome domain in each study were sought (e.g. for all measures, time points, analyses), and if not, the methods used to decide which results to collect. | page 4 |
|  | 10b | List and define all other variables for which data were sought (e.g. participant and intervention characteristics, funding sources). Describe any assumptions made about any missing or unclear information. | page 4 |
| Study risk of bias assessment | 11 | Specify the methods used to assess risk of bias in the included studies, including details of the tool(s) used, how many reviewers assessed each study and whether they worked independently, and if applicable, details of automation tools used in the process. | page 5 |
| Effect measures | 12 | Specify for each outcome the effect measure(s) (e.g., risk ratio, mean difference) used in the synthesis or presentation of results. | page 5 |
| Synthesis methods | 13a | Describe the processes used to decide which studies were eligible for each synthesis (e.g., tabulating the study intervention characteristics and comparing against the planned groups for each synthesis (item #5)). | page 5 |
|  | 13b | Describe any methods required to prepare the data for presentation or synthesis, such as handling of missing summary statistics, or data conversions. | page 5 |
|  | 13c | Describe any methods used to tabulate or visually display the results of individual studies and syntheses. | page 5 |
|  | 13d | Describe any methods used to synthesize results and provide a rationale for the choice(s). If meta-analysis was performed, describe the model(s), method(s) to identify the presence and extent of statistical heterogeneity, and software package(s) used. | page 5 |
|  | 13e | Describe any methods used to explore possible causes of heterogeneity among study results (e.g., subgroup analysis, meta-regression). | page 5 |
|  | 13f | Describe any sensitivity analyses conducted to assess the robustness of the synthesized results. | page 5 |
| Reporting bias assessment | 14 | Describe any methods used to assess the risk of bias due to missing results in a synthesis (arising from reporting biases). | page 5 |
| Certainty assessment | 15 | Describe any methods used to assess certainty (or confidence) in the body of evidence for an outcome. | - |
| **RESULTS** | | |  |
| Study selection | 16a | Describe the results of the search and selection process, from the number of records identified in the search to the number of studies included in the review, ideally using a flow diagram. | page 5 |
|  | 16b | Cite studies that might appear to meet the inclusion criteria, but which were excluded, and explain why they were excluded. | page 5 |
| Study characteristics | 17 | Cite each included study and present its characteristics. | page 5 |
| Risk of bias in studies | 18 | present assessments of risk of bias for each included study. | page 12 |
| Results of individual studies | 19 | For all outcomes, present, for each study: (a) summary statistics for each group (where appropriate) and (b) an effect estimate and its precision (e.g. confidence/credible interval), ideally using structured tables or plots. | page 5-7 |
| Results of syntheses | 20a | For each synthesis, briefly summarise the characteristics and risk of bias among contributing studies. | page 8-12 |
|  | 20b | present results of all statistical syntheses conducted. If meta-analysis was done, present for each the summary estimate and its precision (e.g. confidence/credible interval) and measures of statistical heterogeneity. If comparing groups, describe the direction of the effect. | page 8-12 |
|  | 20c | present results of all investigations of possible causes of heterogeneity among study results. | page 9 |
|  | 20d | present results of all sensitivity analyses conducted to assess the robustness of the synthesized results. | - |
| Reporting biases | 21 | present assessments of risk of bias due to missing results (arising from reporting biases) for each synthesis assessed. | page 11 |
| Certainty of evidence | 22 | present assessments of certainty (or confidence) in the body of evidence for each outcome assessed. | None |
| **DISCUSSION** | | |  |
| Discussion | 23a | provide a general interpretation of the results in the context of other evidence. | page 12-14 |
|  | 23b | Discuss any limitations of the evidence included in the review. | page 14 |
|  | 23c | Discuss any limitations of the review processes used. | None |
|  | 23d | Discuss implications of the results for practice, policy, and future research. | page 14 |
| **OTHER INFORMATION** | | |  |
| Registration and protocol | 24a | provide registration information for the review, including register name and registration number, or state that the review was not registered. | page 3 |
|  | 24b | Indicate where the review protocol can be accessed, or state that a protocol was not prepared. | page 16 |
|  | 24c | Describe and explain any amendments to information provided at registration or in the protocol. | page 3 |
| Support | 25 | Describe sources of financial or non-financial support for the review, and the role of the funders or sponsors in the review. | page 15 |
| Competing interests | 26 | Declare any competing interests of review authors. | page 15 |
| Availability of data, code and other materials | 27 | Report which of the following are publicly available and where they can be found: template data collection forms; data extracted from included studies; data used for all analyses; analytic code; any other materials used in the review. | page 15 |

[**Table S2.**](#__RefHeading___Toc49954750) English databases (PubMed, Embase, Cochrane Library, Web of Science, and Scopus) and

Chinese databases (CNKI, VIP, WanFang, and CBM) Search Strategy (February 12, 2023)

| Database | Search Strategy | Records |
| --- | --- | --- |
| PubMed | (“Systemic inflammation response index” OR “System inflammation response index” OR “Systemic inflammatory response index” OR “SIRI”) AND (“Patients”) [All Fields] | 561+1^※^ |
| Embase | (“Systemic inflammation response index” OR “System inflammation response index” OR “Systemic inflammatory response index” OR “SIRI”) AND (“Patients”) | 1253 |
| Web of Science | (“Systemic inflammation response index” OR “System inflammation response index” OR “Systemic inflammatory response index” OR “SIRI”) AND (“Patients”) [All Fields] | 239 |
| Scopus | (“Systemic inflammation response index” OR “System inflammation response index” OR “Systemic inflammatory response index” OR “SIRI”) AND (“Patients”) | 311 |
| Cochrane Library | (“Systemic inflammation response index” OR “System inflammation response index” OR “Systemic inflammatory response index” OR “SIRI”) AND (“Patients”) | 68 |
| Chinese databases*  (CNKI, WanFang, VIP and CBM) | 全身炎症免疫指数 | 5* |
| Total | | 2437 |

^※^ One article met the criteria identifying from PubMed after February 12, 2023.

* These databases were searched manually, and five articles met the included criteria.

**Table S3. The modified Rankin Scale.**

| Grade | Description |
| --- | --- |
| 0 | No symptoms at all. |
| 1 | No significant disability despite symptoms: able to carry out all usual duties and activities. |
| 2 | Slight disability: unable to cay out all previous activities but able to look after own affairs without assistance. |
| 3 | Moderate disability: requiring some help, but able to walk without assistance. |
| 4 | Moderately severe disability: unable to walk without assistance, and unable to attend to own bodily needs without assistance. |
| 5 | Severe disability: bedridden, incontinent,and requiring constant nursing care and attention. |

mRS 0-2: good outcome; good clinical outcome; favorable outcome; functional independence; good functional outcome.

mRS 3-5: poor outcome; poor clinical outcome; unfavorable outcome; functional dependence; poor functional outcome.

The Glasgow Outcome Scale

| Grade | Name | Description |
| --- | --- | --- |
| 1 | Dead | - |
| 2 | Vegetative state | Unable to interact with environment; unresponsive |
| 3 | Severe disability | Able to follow commands, unable to live independently. |
| 4 | Moderate disability | Able to live independently; unable to return to work or school. |
| 5 | Good recovery | Able to return to work or school. |

GOS 1-3: poor outcome; poor clinical outcome; unfavorable outcome; functional dependence; poor functional outcome.

GOS 4-5: good outcome; good clinical outcome; favorable outcome; functional independence; good functional outcome.

[**Table S4.** ROB assessment for the quality of studies in meta-analysis](#__RefHeading___Toc49954757) via NOS Scale.

| Study | Year | Selection | | | | Comparability | | Exposure | | | Stars |
| --- | --- | --- | --- | --- | --- | --- | --- | --- | --- | --- | --- |
|  |  | Q1 | Q2 | Q3 | Q4 | Q5 | Q6 | Q7 | Q8 | Q9 |  |
| Fei et al | 2020 | * | * | * | - | - | * | * | - | - | 5 |
| Zhang et al | 2020 | * | * | * | - | * | * | * | * | - | 7 |
| Zhang Z et al | 2020 | * | * | * | - | - | * | * | - | - | 5 |
| Lattanzi et al | 2021 | * | * | * | - | * | * | * | * | * | 8 |
| Li et al | 2021 | * | * | * | - | * | * | * | * | * | 8 |
| Shi et al | 2021 | * | * | * | - | * | * | * | * | * | 8 |
| Yi et al | 2021 | * | * | * | - | * | * | * | * | * | 8 |
| Yun et al | 2021 | * | * | * | - | * | * | * | * | * | 8 |
| Zhang et al | 2021 | * | * | * | - | - | * | * | * | * | 7 |
| Li et al | 2022 | * | * | * | - | * | * | * | * | * | 8 |
| Ma et al | 2022 | * | * | * | * | * | * | * | * | * | 9 |
| Wang et al | 2022 | * | * | * | * | - | * | * | - | * | 7 |
| Yu et al | 2022 | * | * | * | - | * | * | * | * | * | 8 |
| Zhou et al | 2022 | * | * | * | * | * | * | * | * | * | 9 |
| Dang et al | 2023 | * | * | * | - | - | * | * | * | * | 7 |
| Huang et al | 2023 | * | * | * | - | * | * | * | - | * | 7 |
| Wang JJ et al | 2023 | * | * | * | * | - | * | * | * | * | 8 |
| Yan et al | 2023 | * | * | * | - | - | * | * | - | * | 6 |
| Yu et al | 2023 | * | * | * | * | - | * | * | - | * | 7 |
| Chu et al | 2023 | * | * | * | - | * | * | * | * | * | 8 |
| Hou et al | 2023 | * | * | * | * | * | * | * | * | * | 9 |
| Wang RH et al | 2023 | * | * | * | * | * | * | * | * | * | 9 |

Q1 Is the case definition adequate?

Q2 Representativeness of the cases.

Q3 Selection of Controls.

Q4 Definition of Controls.

Q5 study controls for the most important factor.

Q6 study controls for any additional factor.

Q7 Ascertainment of exposure.

Q8 Same method of ascertainment for cases and controls.

Q9 Non-Response rate.

**Figure S1.** Funnel plot results of main end points.

SIRI between poor outcome and good outcome SIRI for poor outcome **(**dichotomous variable**)**

**
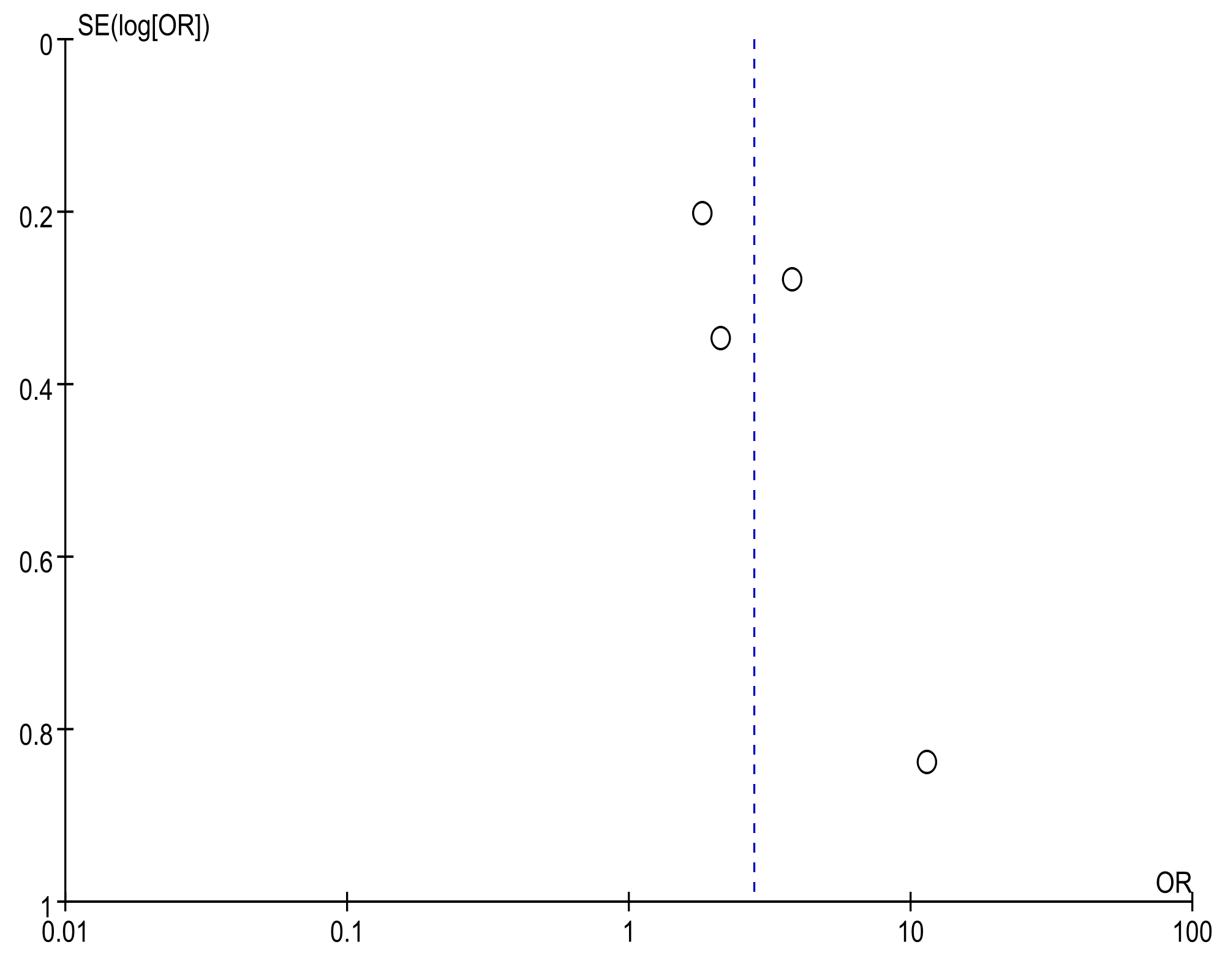

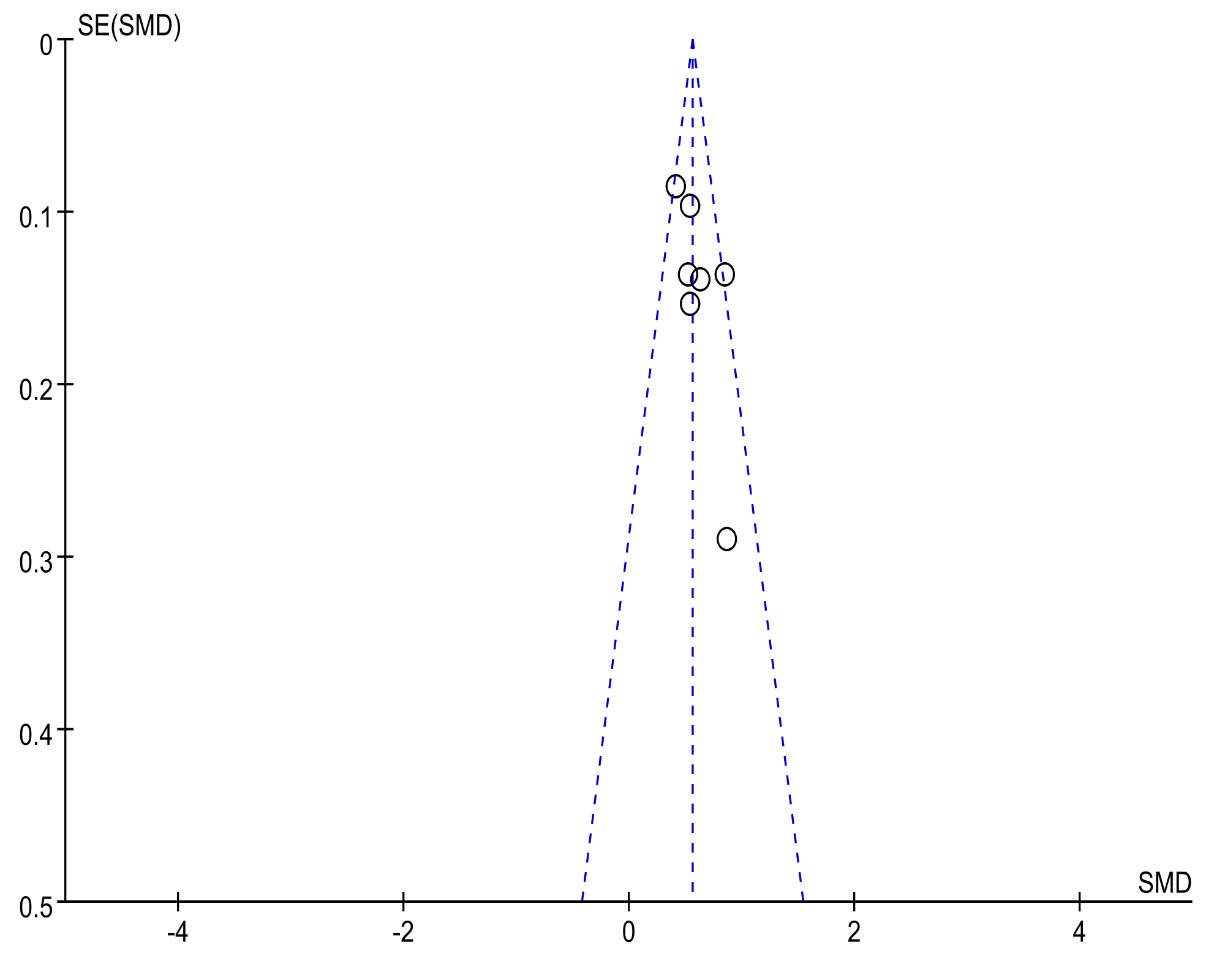
**

SIRI for poor outcome (continuous variable) SIRI predictive value of poor outcome

**
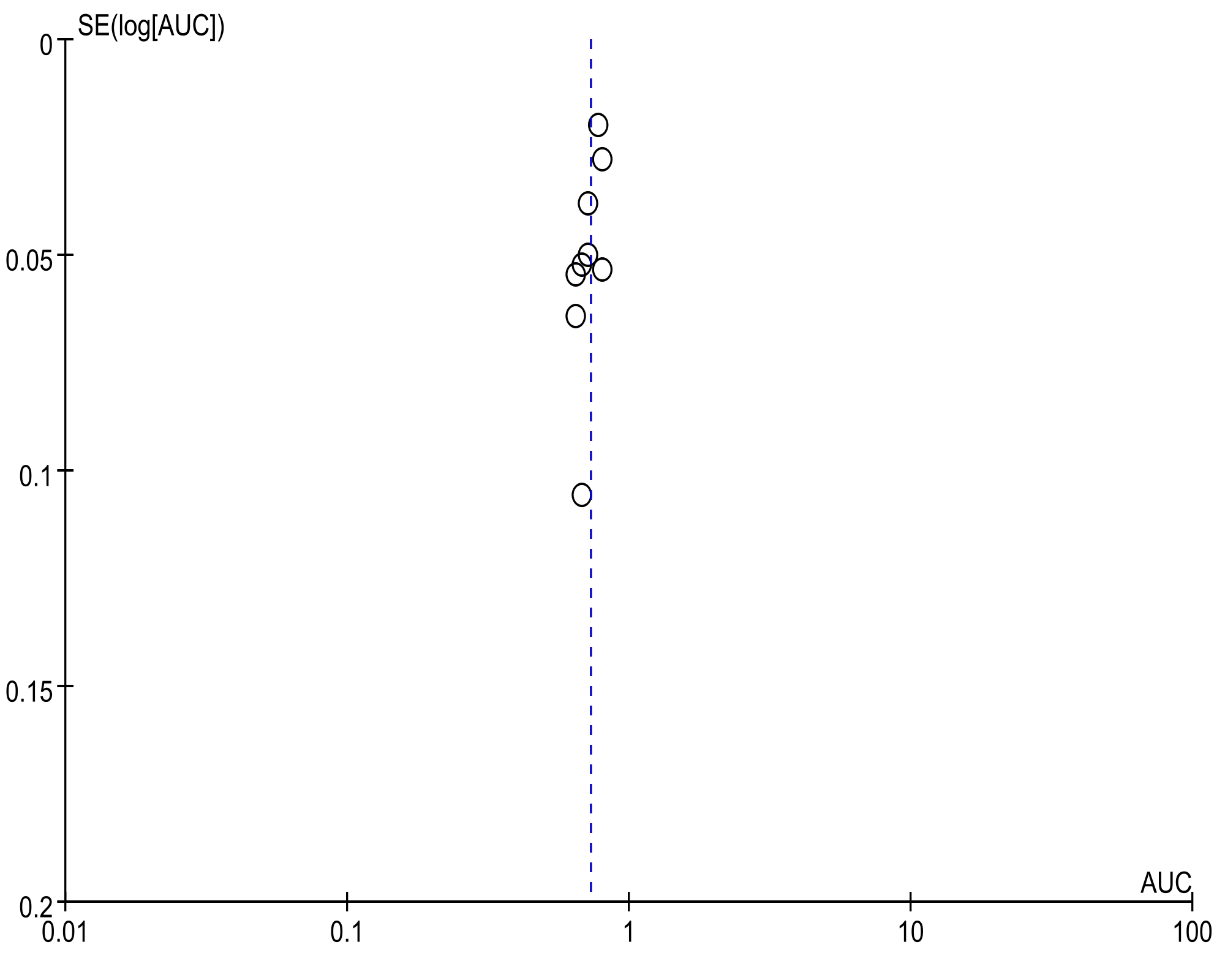

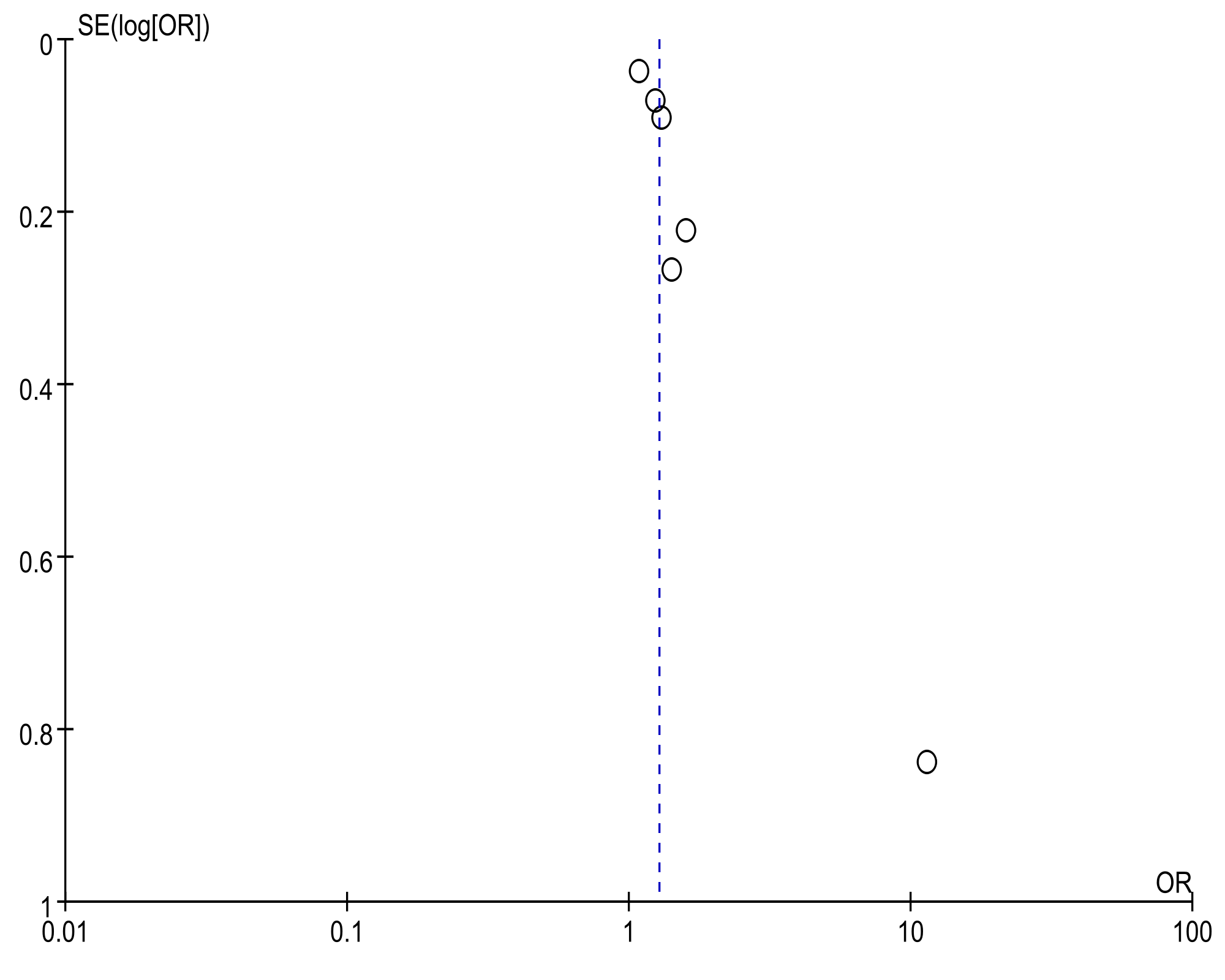
**

SIRI between SAP and Non-SAP SIRI for SAP (continuous variable)

**
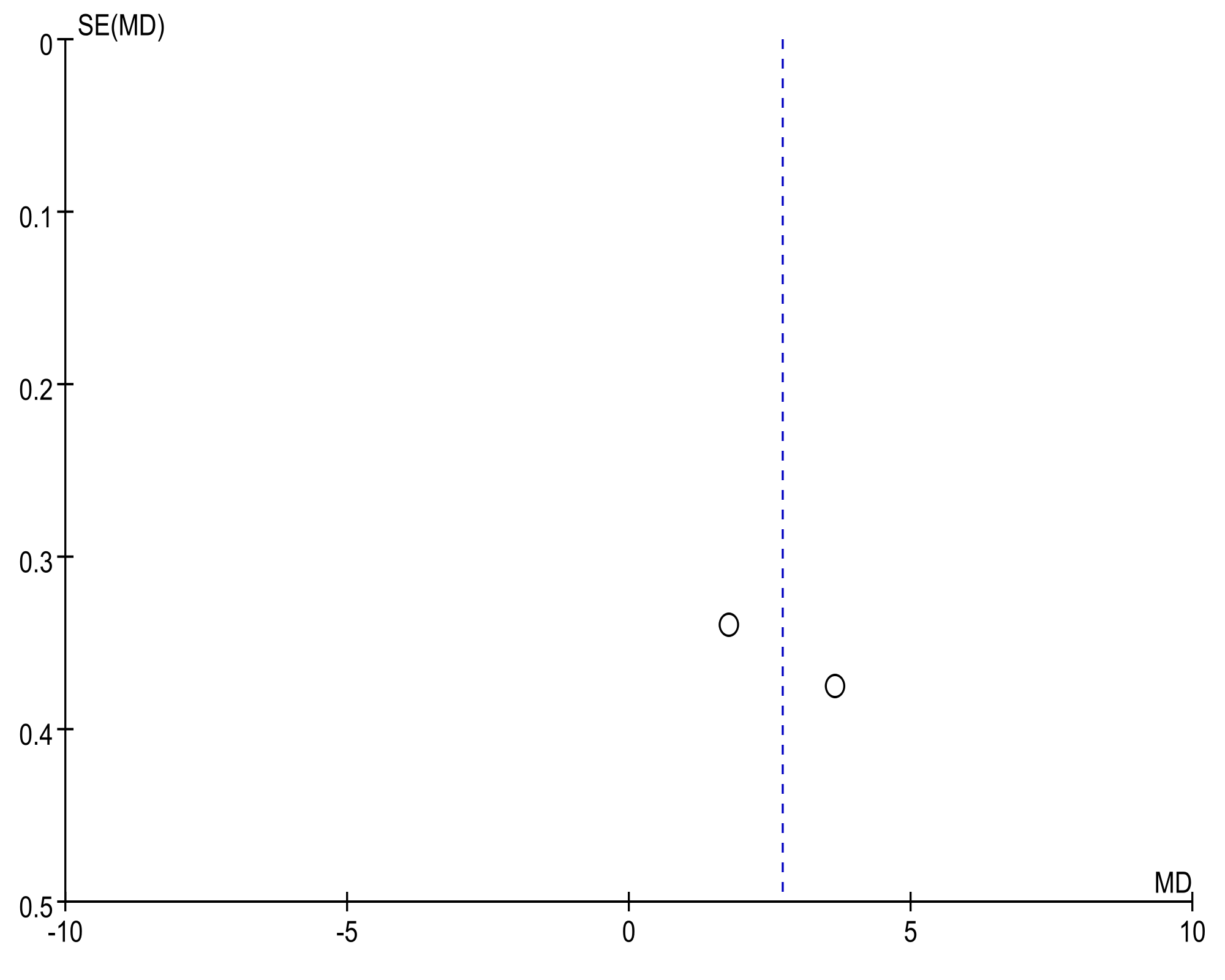

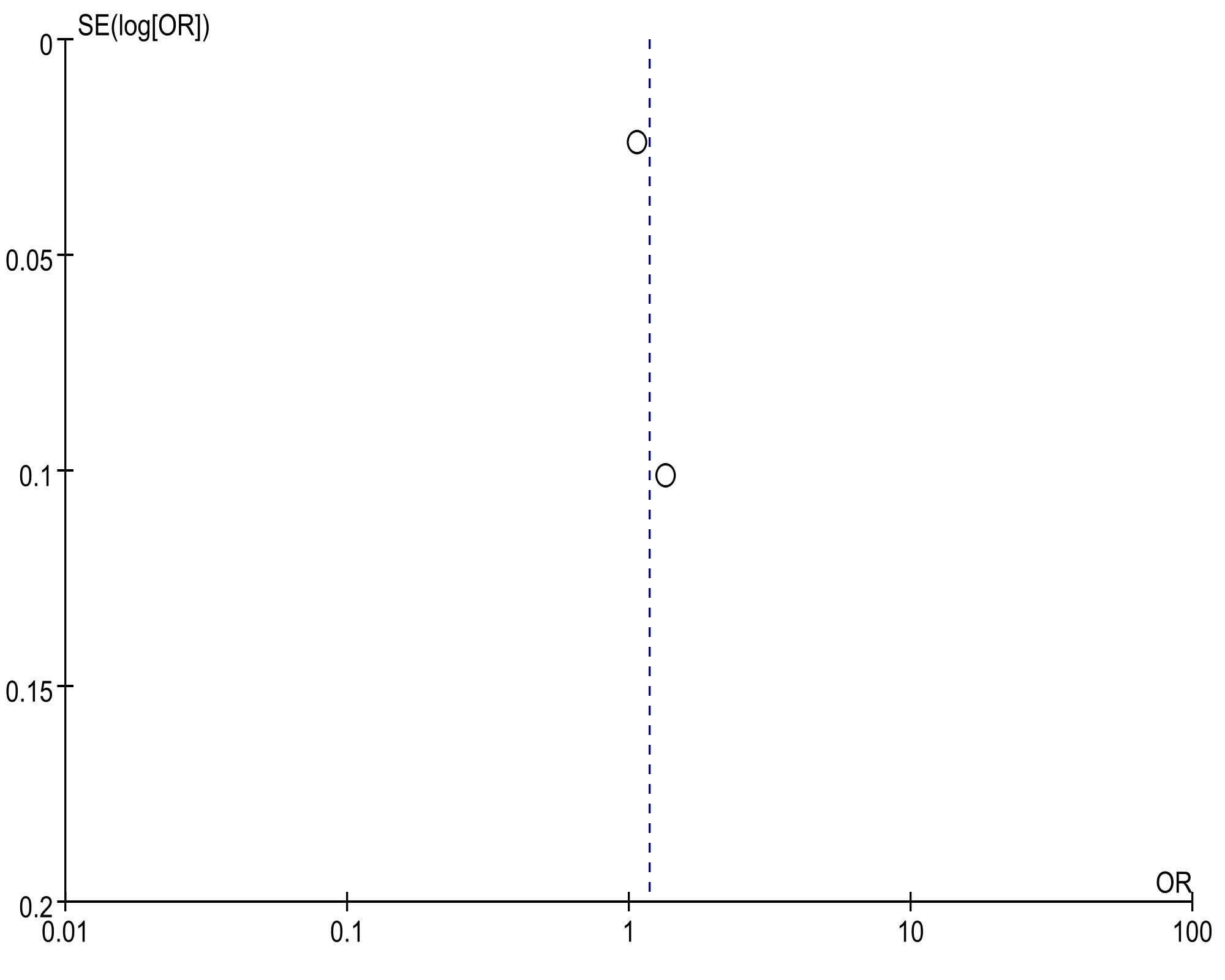
**

SIRI for SAP **(**dichotomous variable**)** SIRI predictive value of SAP

**
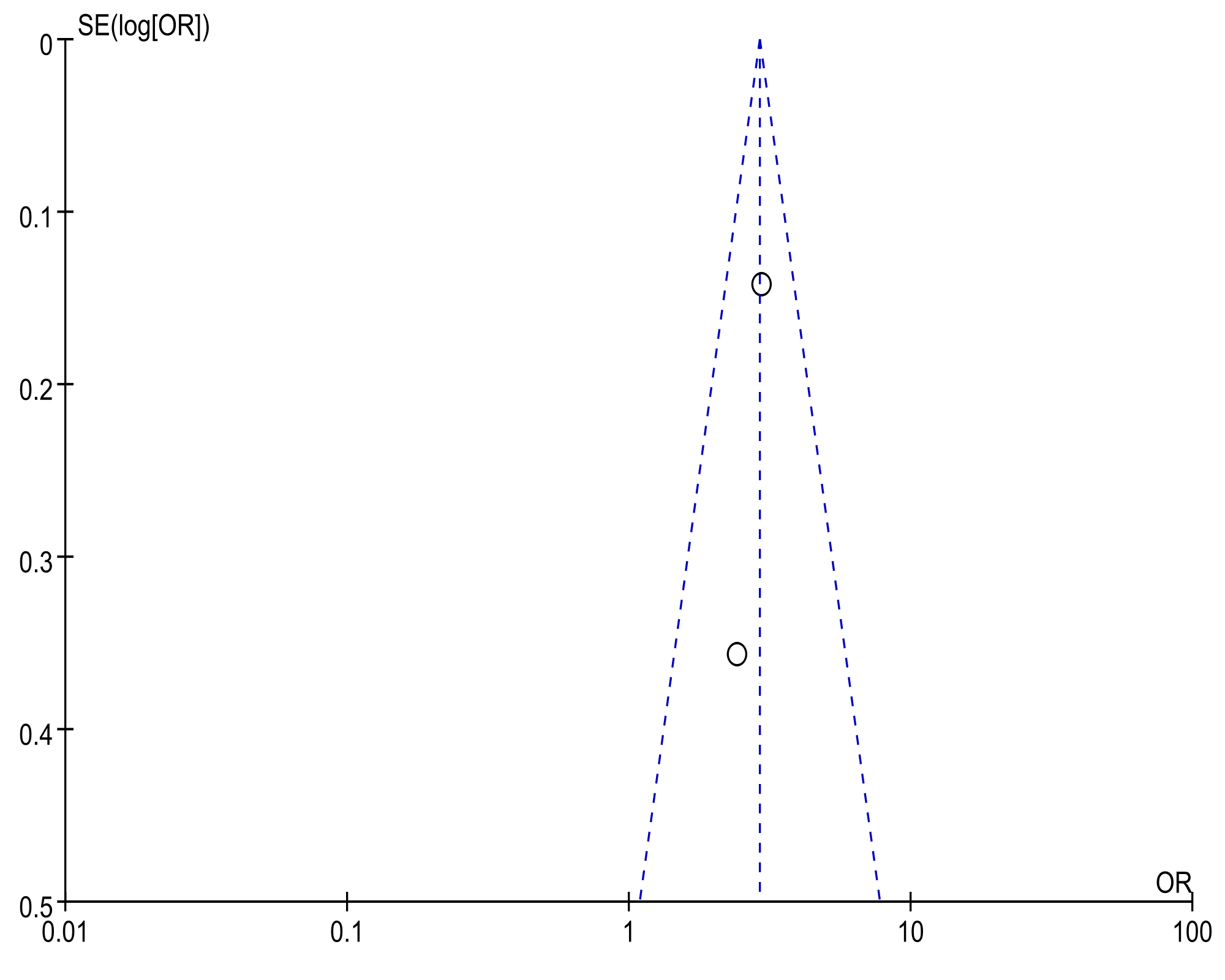

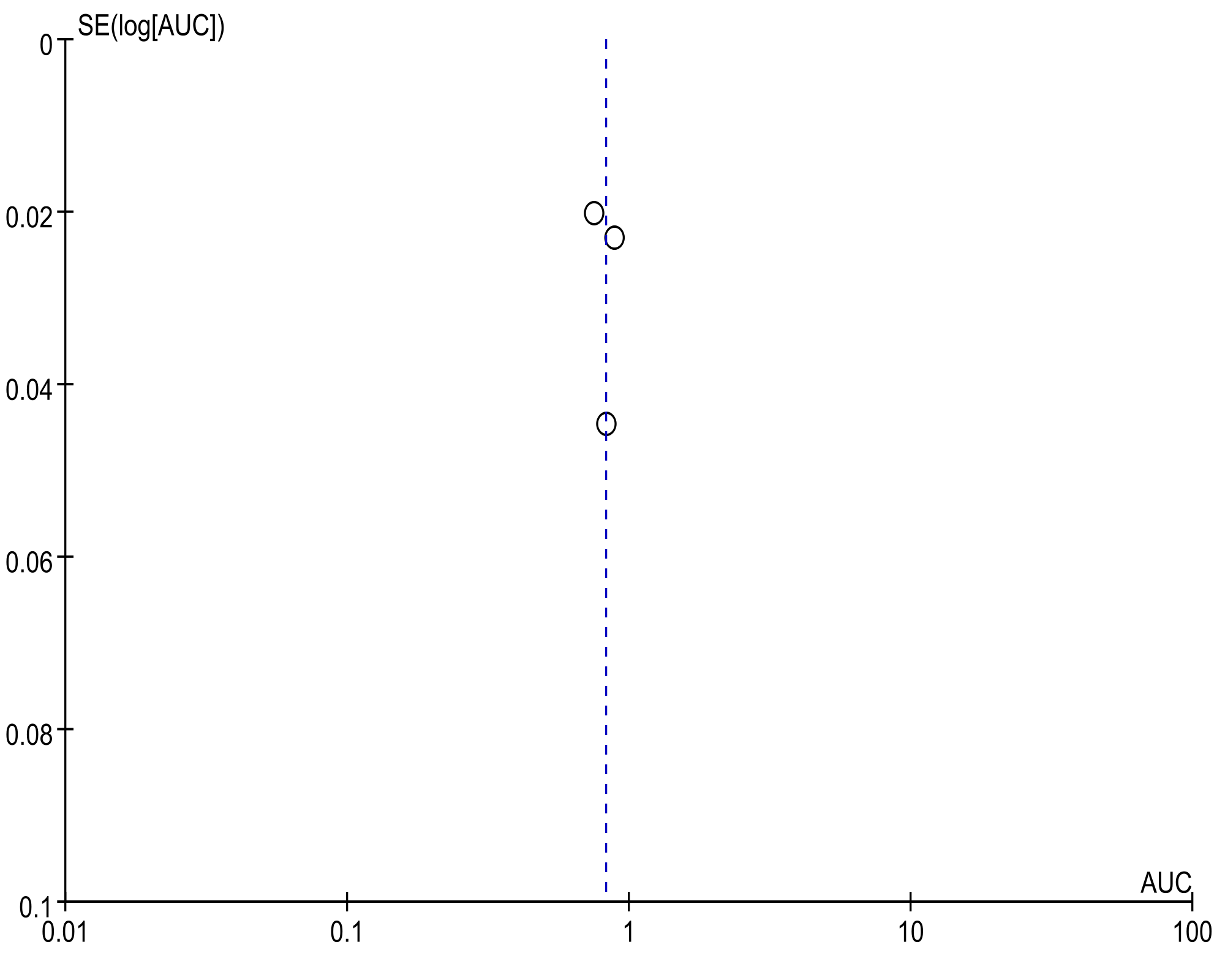
**

SIRI for in-hospital mortality SIRI for 1-month mortality

**
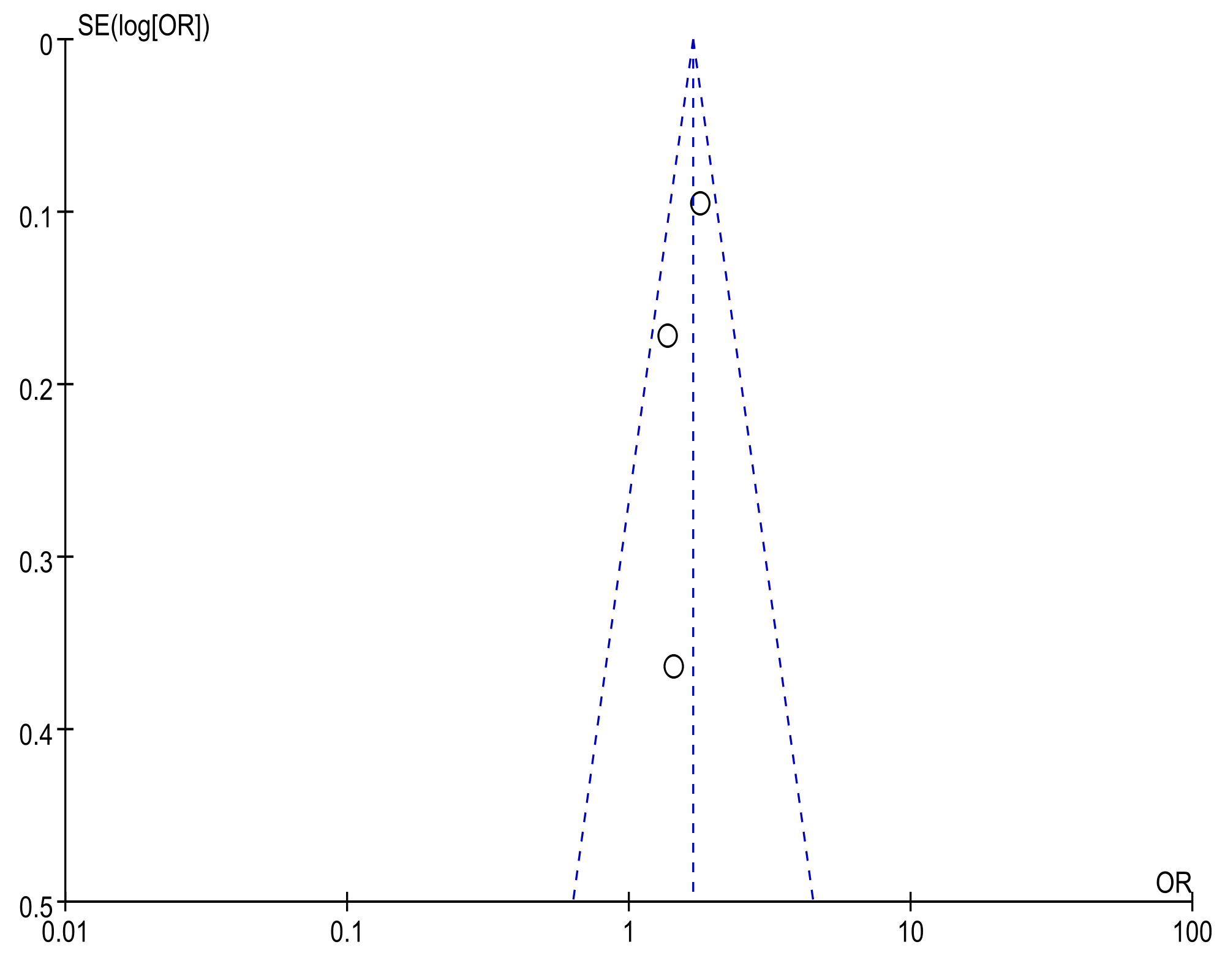

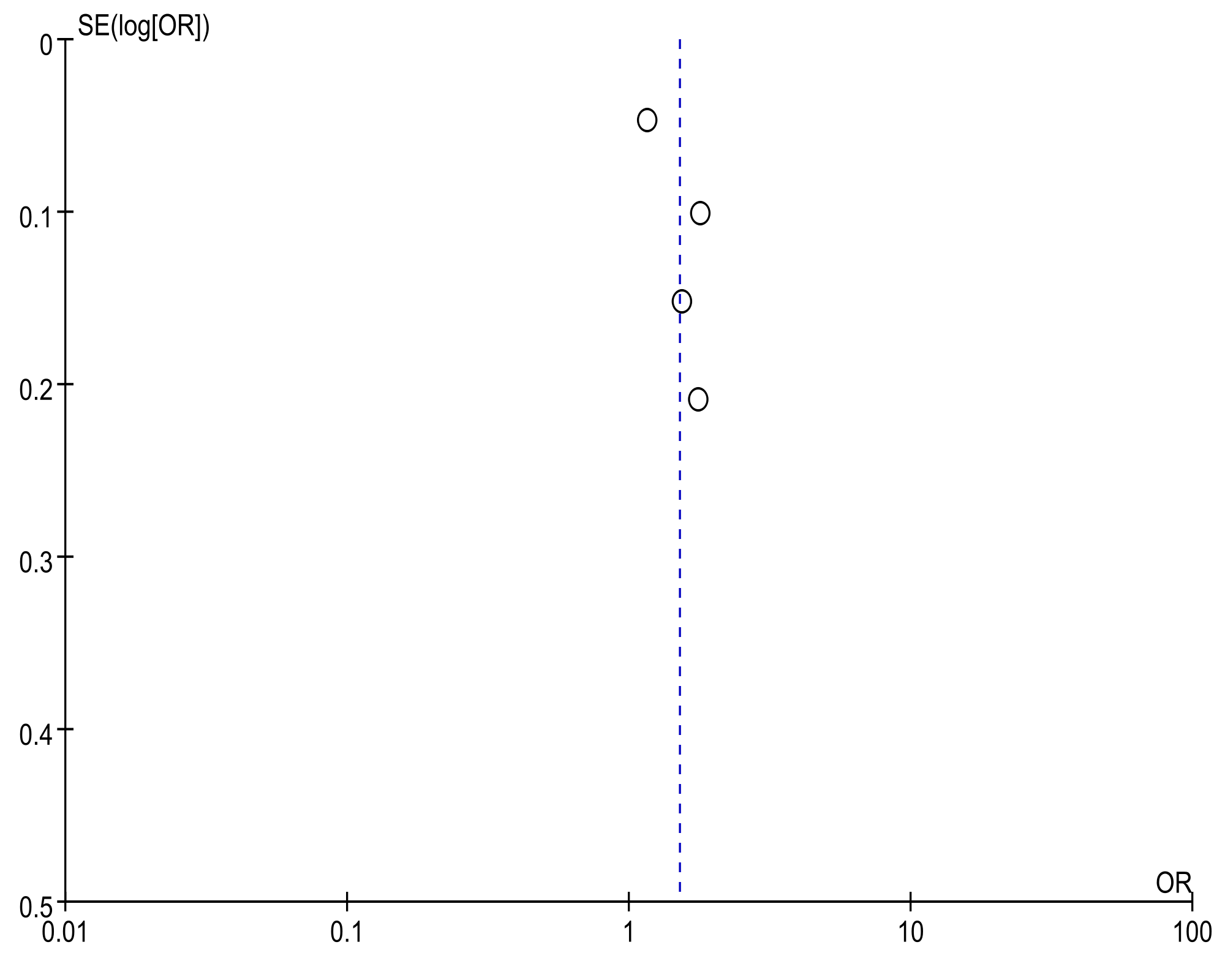
**

SIRI for 3-months mortality SIRI for 3-months mortality

**
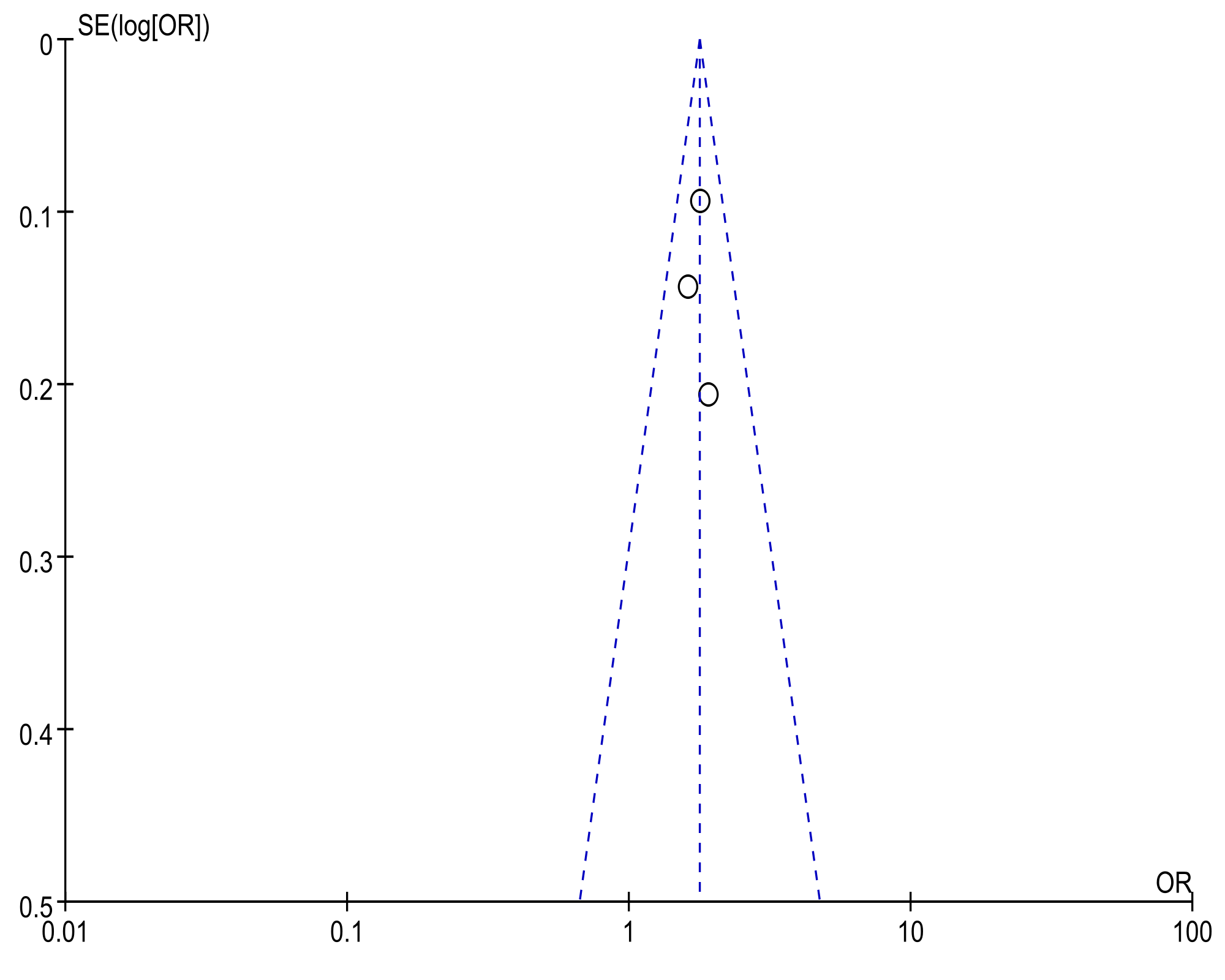

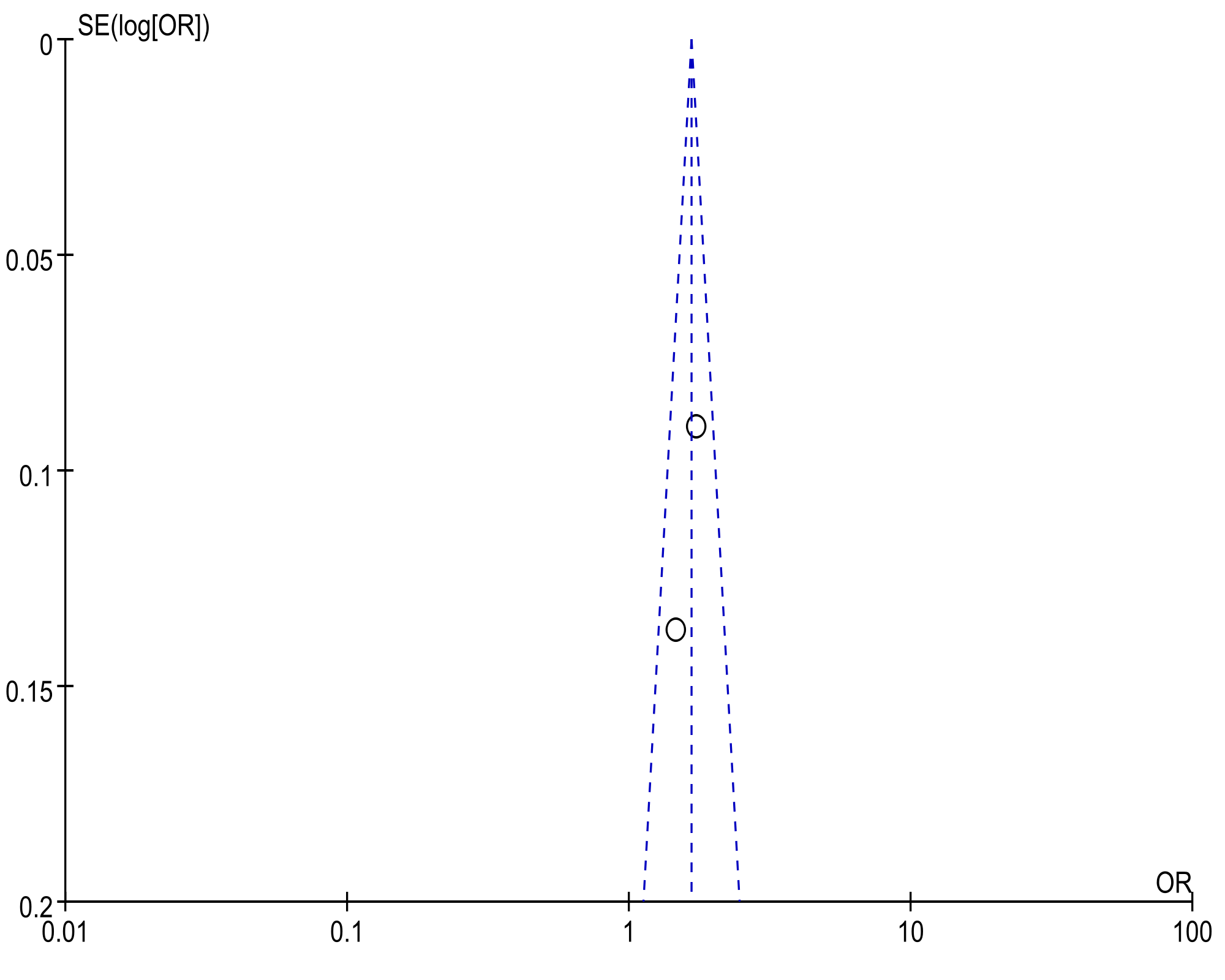
**

SIRI for HHS SIRI for mFS

**
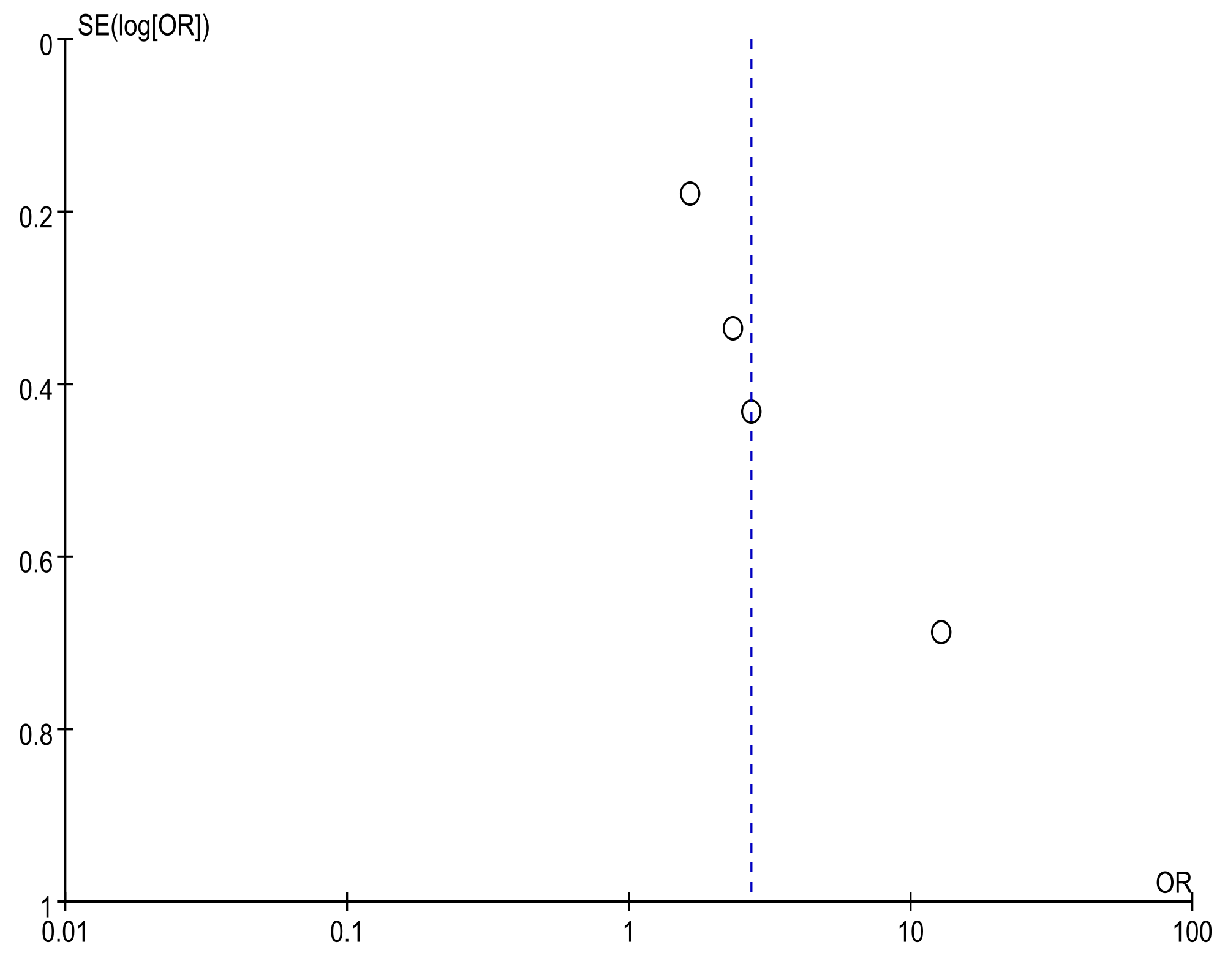

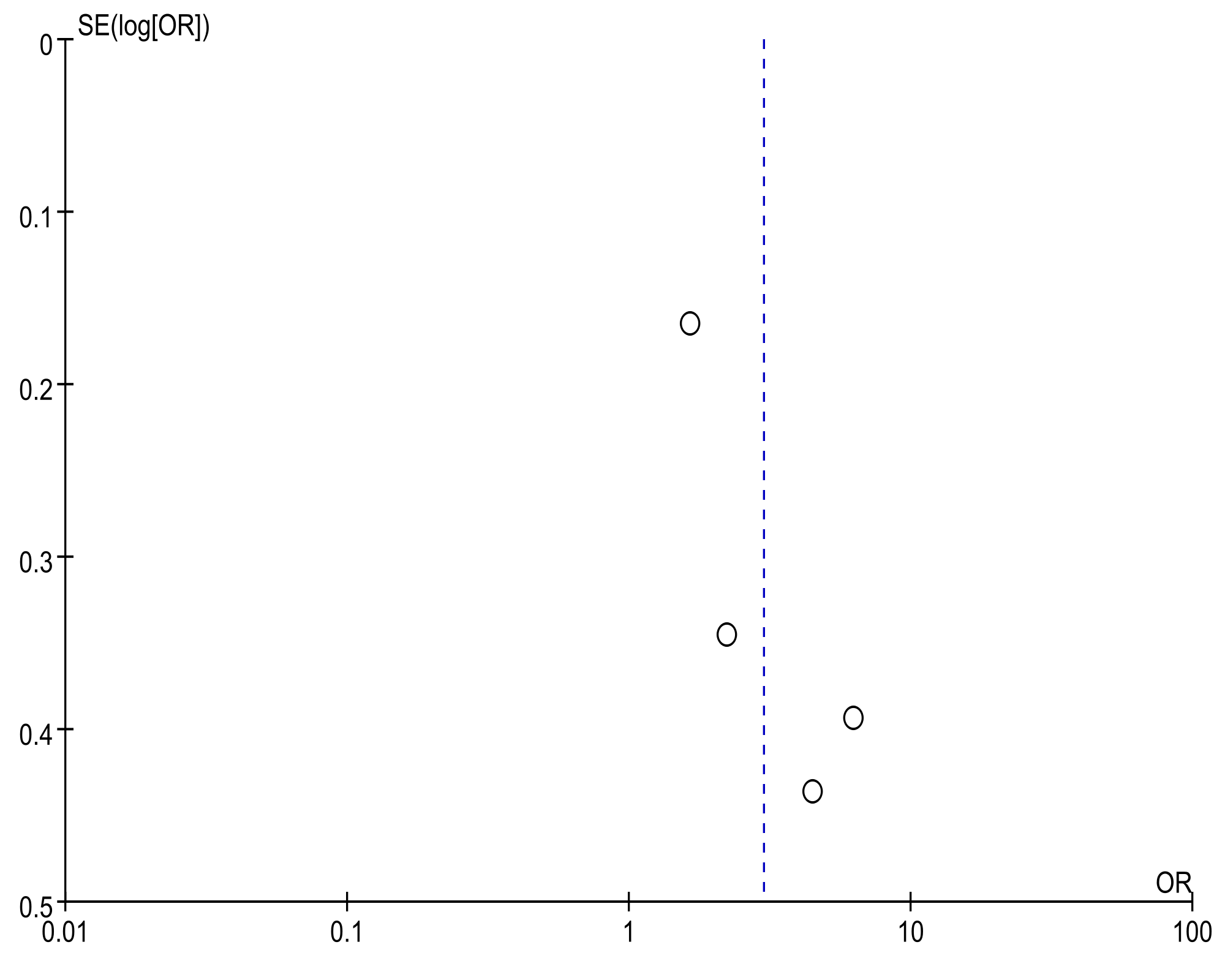
**

SIRI for DCI SIRI for AHC

**
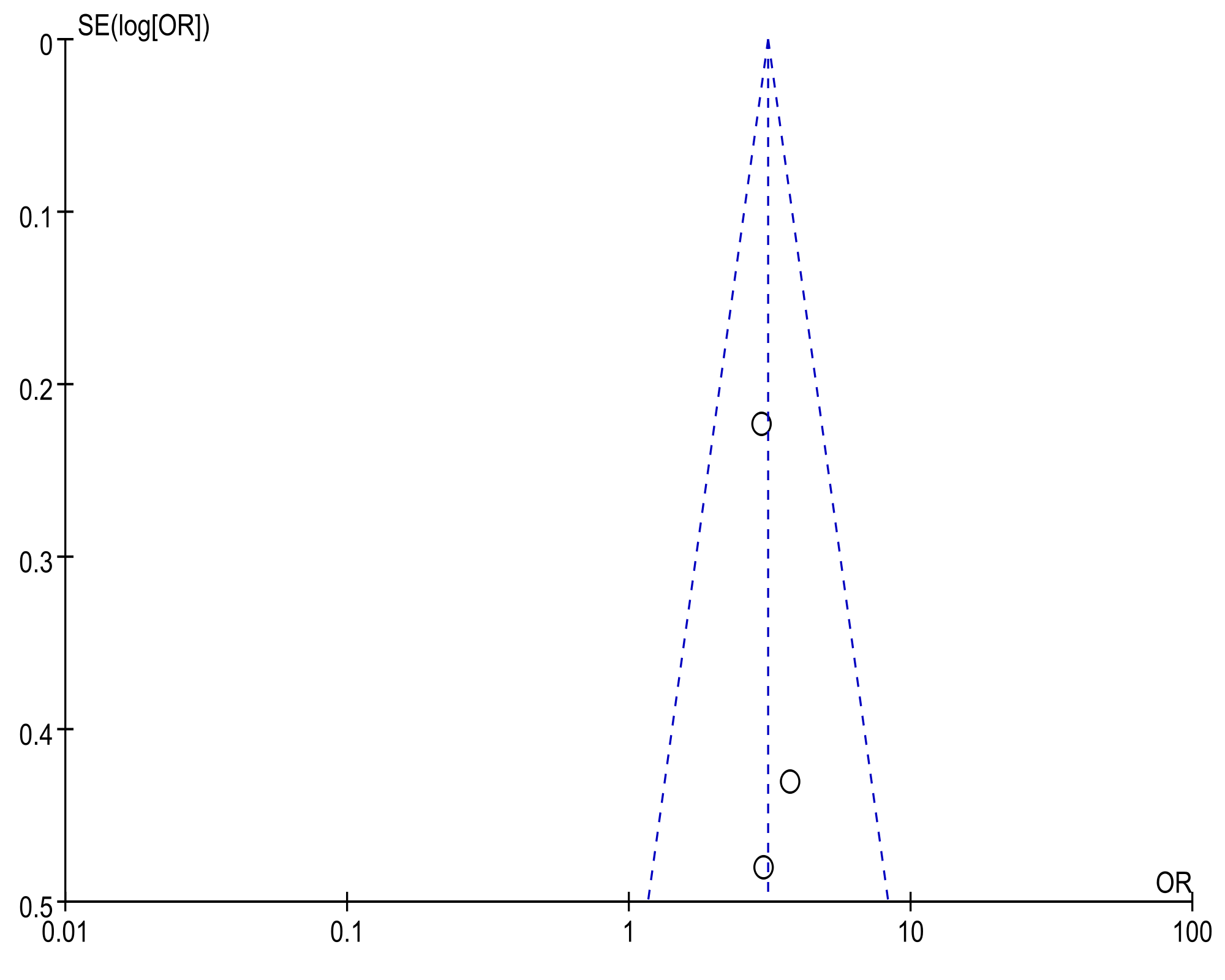

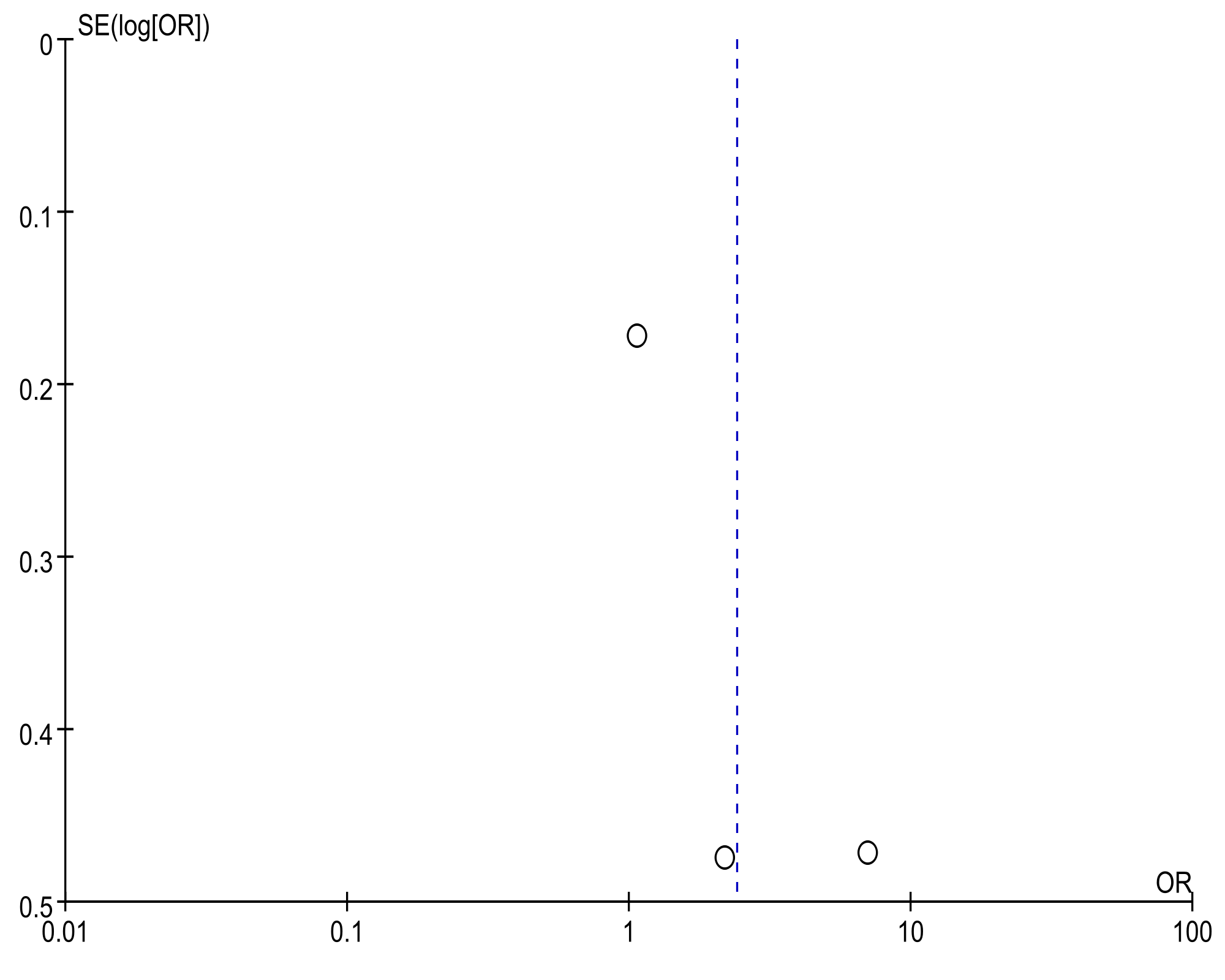
**

SIRI for vasospasm

**
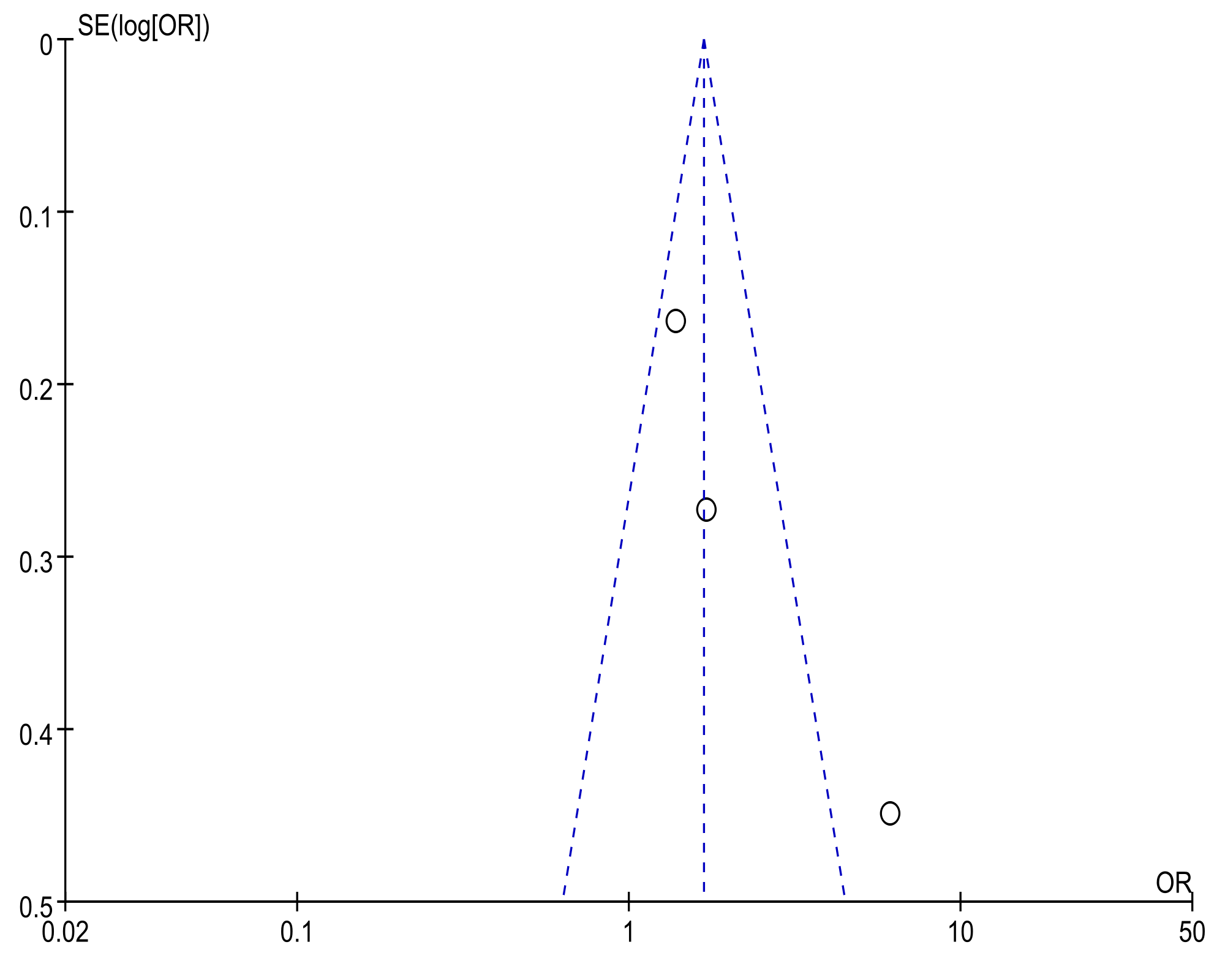
**

Subgroup analyses of sub-type of stroke

**
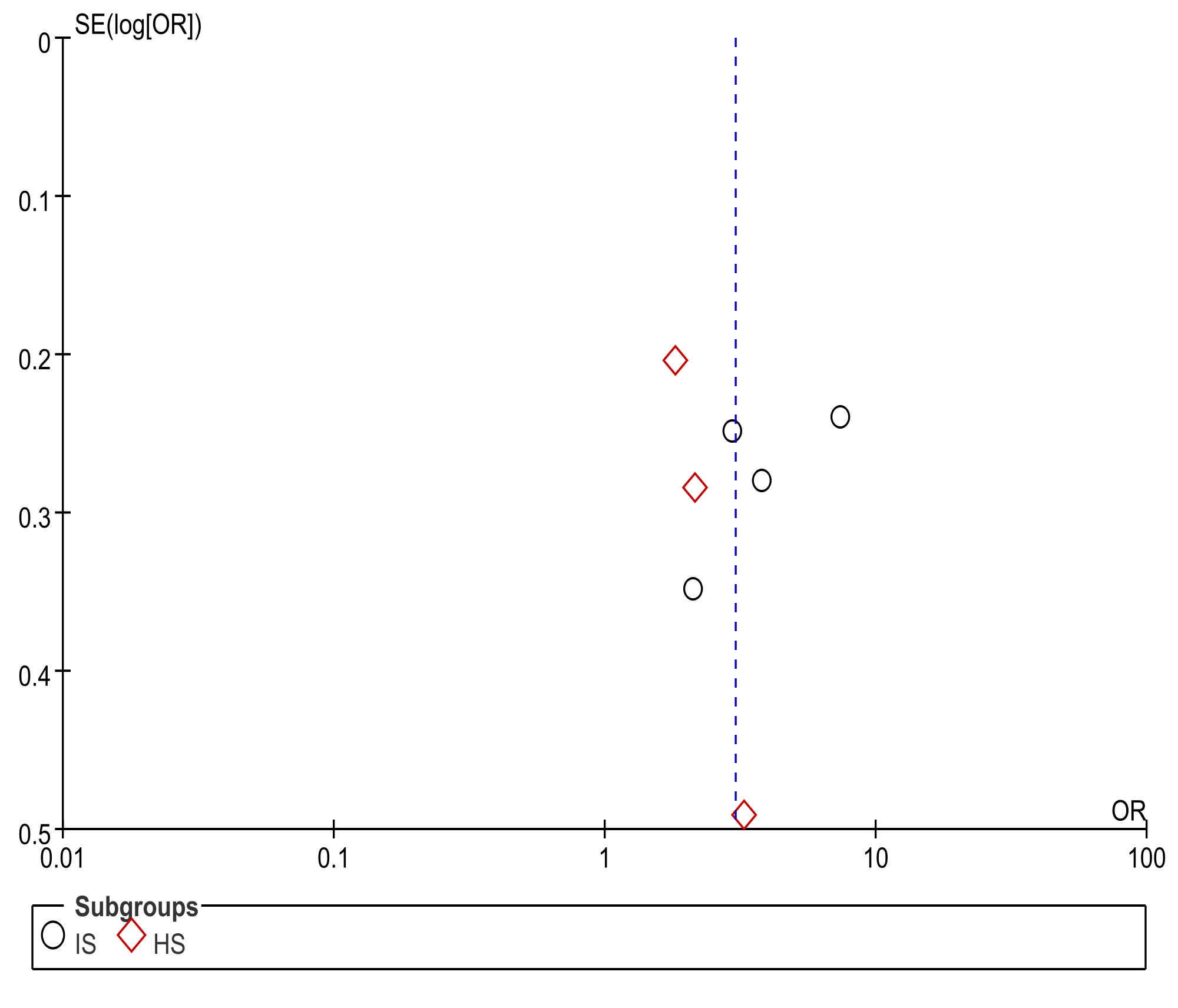

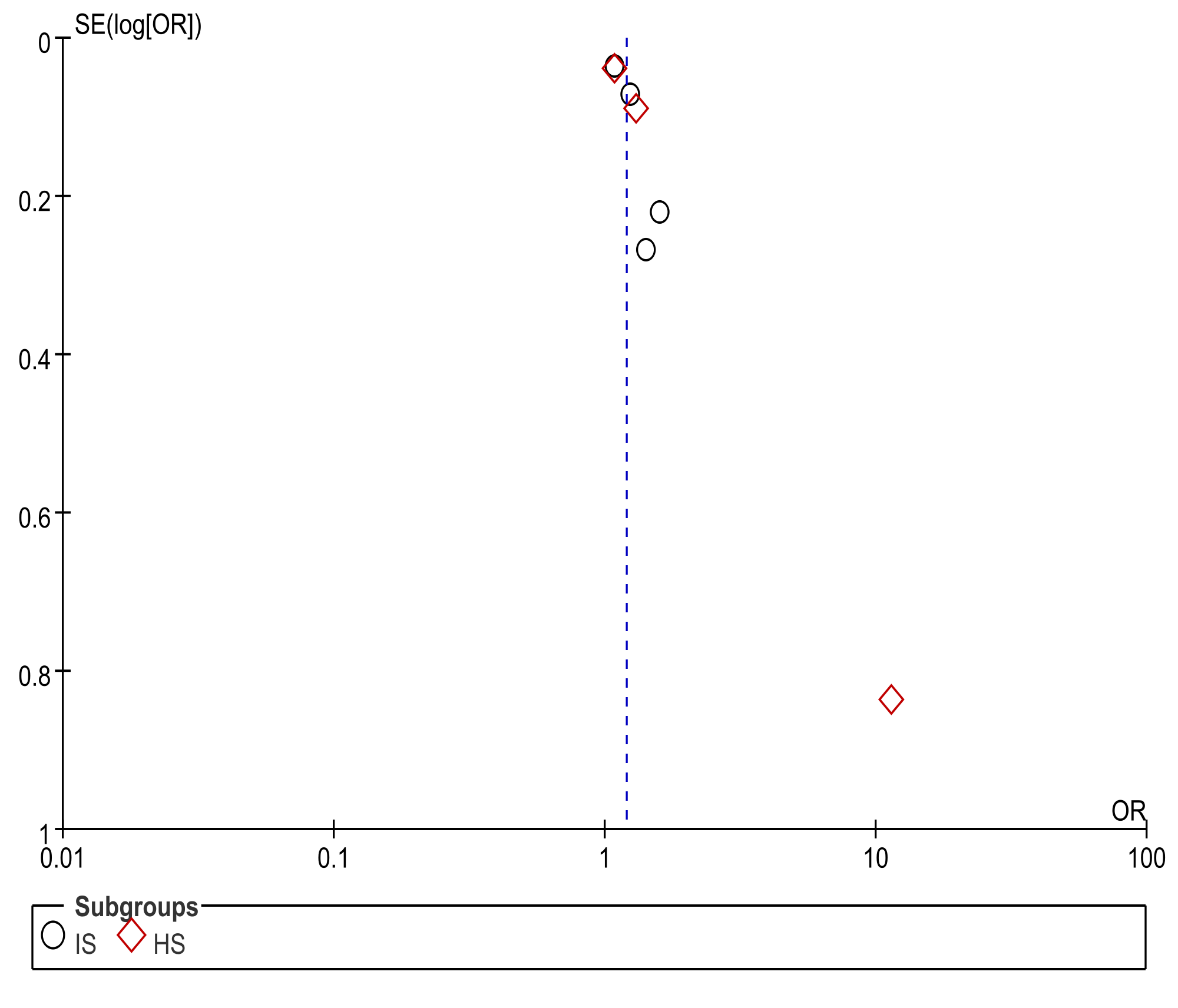

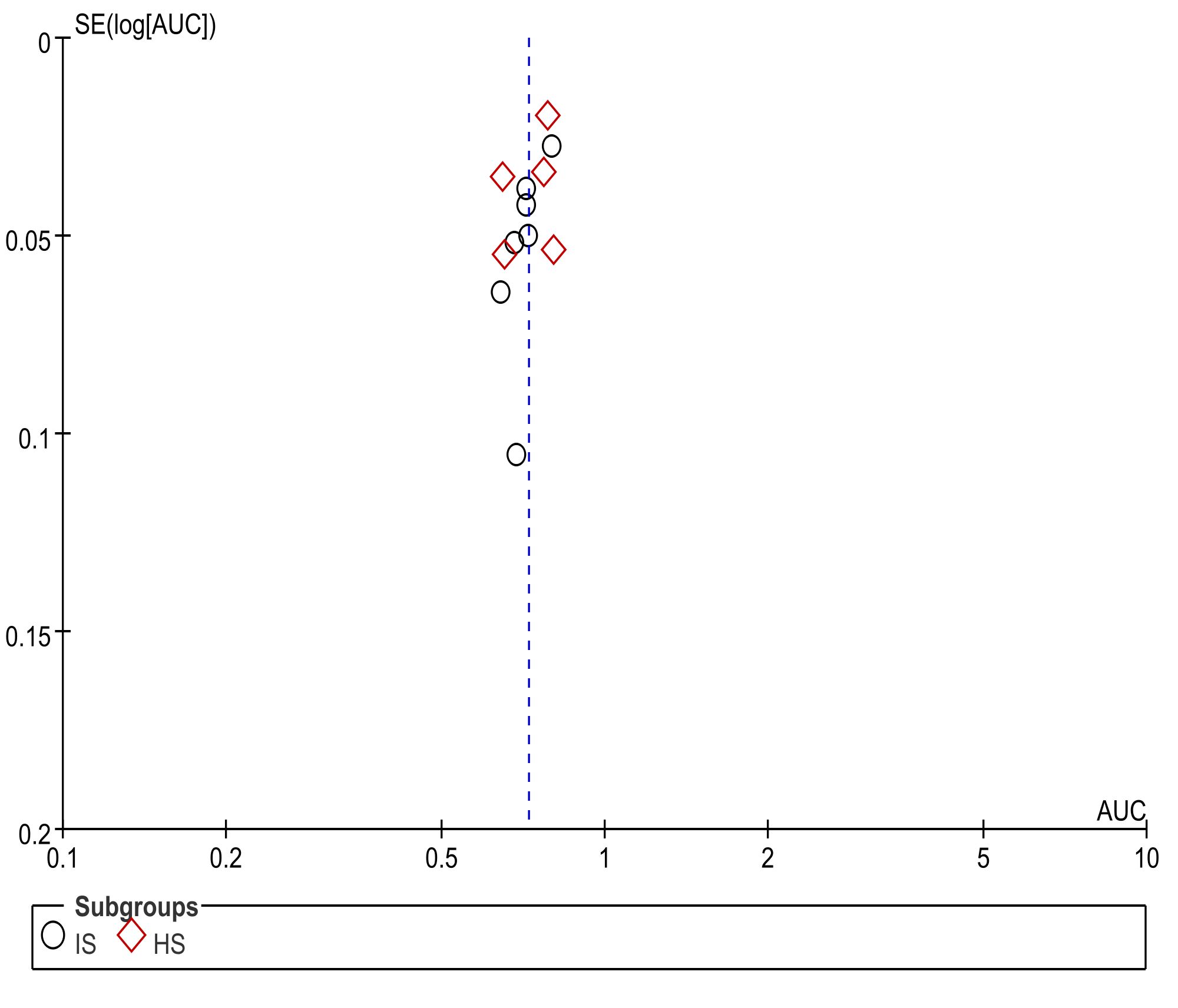

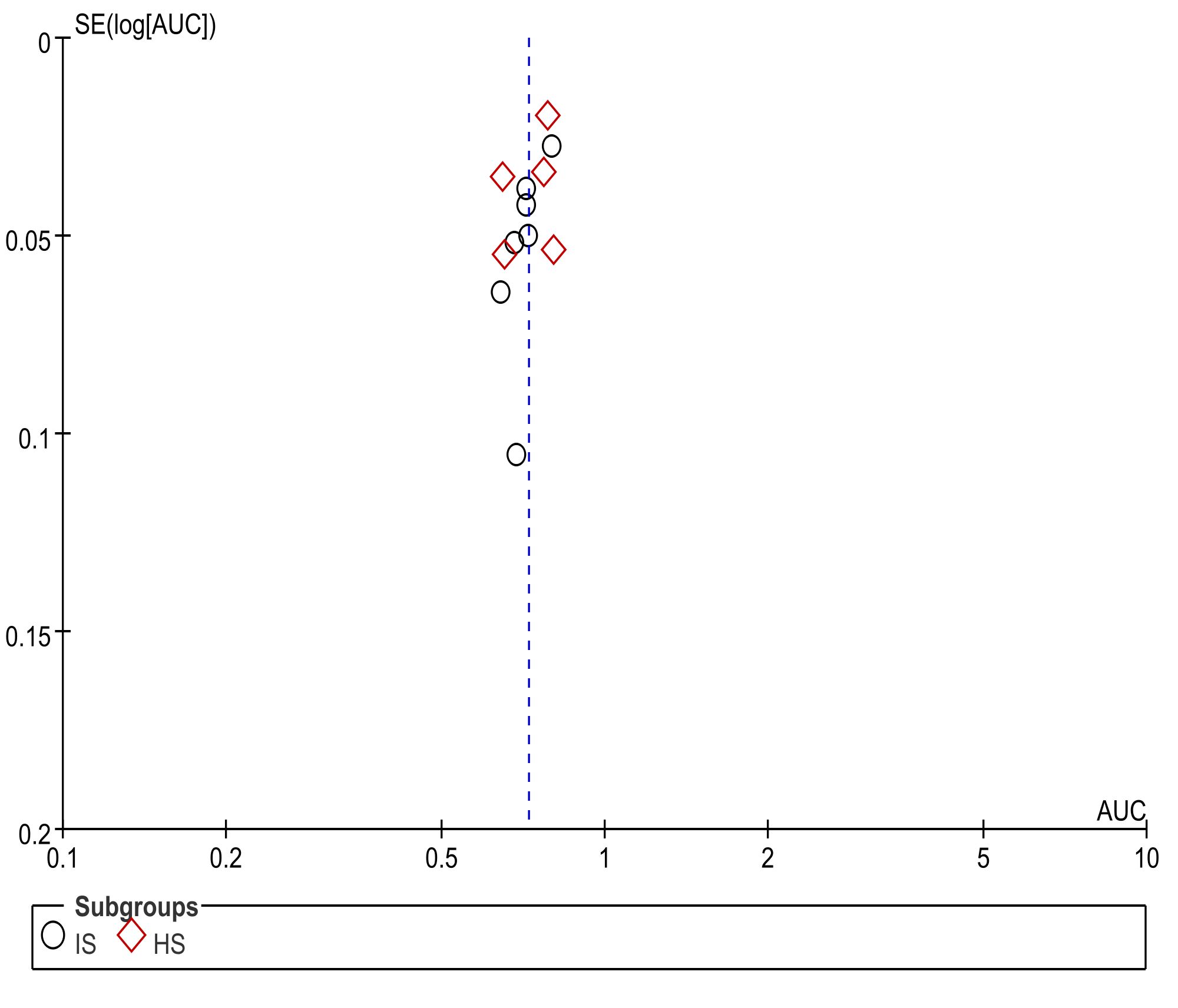
**
